# Macrophage activation syndrome in systemic lupus erythematous and Still’s disease: distinct phenotypes shaped by the underlying diseases

**DOI:** 10.1093/rheumatology/keag490

**Published:** 2026-09-11

**Authors:** Antonio Tonutti, Ilaria Cavazzana, Maria Gerosa, Francesco Caso, Maria De Santis, Francesca Trunfio, Andrew Barr, Vishal Kakkar, Kerem Abacar, Aamir Aslam, Letizia Pia Di Corcia, Tom Macleod, Ala Altaie, Dina Youssef, Md Yuzaiful Md Yusof, Sinisa Savic, Micaela Fredi, Francesca Crisafulli, Micol Frassi, Antonio Vitale, Addolorata Corrado, Franco Franceschini, Carlo Selmi, Roberto Giacomelli, Edward Vital, Piero Ruscitti, Dennis McGonagle

**Affiliations:** Leeds Institute of Rheumatic and Musculoskeletal Medicine, University of Leeds, Leeds, UK; Department of Biomedical Sciences, Humanitas University, Milan, Italy; Rheumatology and Clinical Immunology, IRCCS Humanitas Research Hospital, Milan, Italy; Rheumatology and Clinical Immunology Unit and Department of Clinical and Experimental Sciences, ERN ReCONNET, ASST Spedali Civili and University of Brescia, Brescia, Italy; Department of Clinical Sciences and Community Health, Dipartimento di Eccellenza 2023-2027, University of Milan, Milan, Italy; Unit of Clinical Rheumatology, ASST Gaetano Pini—CTO, Milan, Italy; Rheumatology Research Section, Department of Clinical Medicine and Surgery, University of Naples Federico II, Naples, Italy; ImmunoPharmaLab, Department of Pharmacy, School of Medicine and Surgery, University of Naples Federico II, Naples, Italy; Department of Biomedical Sciences, Humanitas University, Milan, Italy; Rheumatology and Clinical Immunology, IRCCS Humanitas Research Hospital, Milan, Italy; Leeds Institute of Rheumatic and Musculoskeletal Medicine, University of Leeds, Leeds, UK; Rheumatology and Clinical Immunology, Department of Medicine, School of Medicine, University of Rome “Campus Bio-Medico”, Rome, Italy; NIHR Leeds Biomedical Research Centre, The Leeds Teaching Hospitals NHS Trust, Leeds, UK; Leeds Institute of Rheumatic and Musculoskeletal Medicine, University of Leeds, Leeds, UK; NIHR Leeds Biomedical Research Centre, The Leeds Teaching Hospitals NHS Trust, Leeds, UK; Leeds Institute of Rheumatic and Musculoskeletal Medicine, University of Leeds, Leeds, UK; NIHR Leeds Biomedical Research Centre, The Leeds Teaching Hospitals NHS Trust, Leeds, UK; Leeds Institute of Rheumatic and Musculoskeletal Medicine, University of Leeds, Leeds, UK; NIHR Leeds Biomedical Research Centre, The Leeds Teaching Hospitals NHS Trust, Leeds, UK; Leeds Institute of Rheumatic and Musculoskeletal Medicine, University of Leeds, Leeds, UK; Rheumatology and Clinical Immunology, Department of Medicine, School of Medicine, University of Rome “Campus Bio-Medico”, Rome, Italy; Leeds Institute of Rheumatic and Musculoskeletal Medicine, University of Leeds, Leeds, UK; Leeds Institute of Rheumatic and Musculoskeletal Medicine, University of Leeds, Leeds, UK; Department of Rheumatology, Bradford Teaching Hospitals NHS Foundation Trust, Bradford, UK; Leeds Institute of Rheumatic and Musculoskeletal Medicine, University of Leeds, Leeds, UK; NIHR Leeds Biomedical Research Centre, The Leeds Teaching Hospitals NHS Trust, Leeds, UK; Leeds Institute of Rheumatic and Musculoskeletal Medicine, University of Leeds, Leeds, UK; NIHR Leeds Biomedical Research Centre, The Leeds Teaching Hospitals NHS Trust, Leeds, UK; Rheumatology and Clinical Immunology Unit and Department of Clinical and Experimental Sciences, ERN ReCONNET, ASST Spedali Civili and University of Brescia, Brescia, Italy; Rheumatology and Clinical Immunology Unit and Department of Clinical and Experimental Sciences, ERN ReCONNET, ASST Spedali Civili and University of Brescia, Brescia, Italy; Rheumatology and Clinical Immunology Unit and Department of Clinical and Experimental Sciences, ERN ReCONNET, ASST Spedali Civili and University of Brescia, Brescia, Italy; Department of Medical Sciences, Surgery and Neurosciences, Research Center of Systemic Autoinflammatory Diseases and Behçet’s Disease Clinic University of Siena, Siena, Italy; European Reference Network (ERN) for Rare Immunodeficiency, Autoinflammatory and Autoimmune Diseases (RITA) Center, Azienda Ospedaliero-Universitaria Senese, Siena, Italy; Rheumatology Clinic, Department of Medical and Surgical Sciences, University of Foggia, Foggia, Italy; Rheumatology and Clinical Immunology Unit and Department of Clinical and Experimental Sciences, ERN ReCONNET, ASST Spedali Civili and University of Brescia, Brescia, Italy; Department of Biomedical Sciences, Humanitas University, Milan, Italy; Rheumatology and Clinical Immunology, IRCCS Humanitas Research Hospital, Milan, Italy; Rheumatology and Clinical Immunology, Department of Medicine, School of Medicine, University of Rome “Campus Bio-Medico”, Rome, Italy; Leeds Institute of Rheumatic and Musculoskeletal Medicine, University of Leeds, Leeds, UK; NIHR Leeds Biomedical Research Centre, The Leeds Teaching Hospitals NHS Trust, Leeds, UK; Department of Biotechnological and Applied Clinical Sciences, University of L’Aquila, L’Aquila, Italy; Leeds Institute of Rheumatic and Musculoskeletal Medicine, University of Leeds, Leeds, UK; NIHR Leeds Biomedical Research Centre, The Leeds Teaching Hospitals NHS Trust, Leeds, UK

**Keywords:** hemophagocytic lymphohistiocytosis, SLE, Still’s disease, Coombs test, haemolysis, IL-1, clinical features, 18-FDG PET/CT

## Abstract

**Objectives:**

Macrophage activation syndrome (MAS) is a severe complication of Still’s disease (SD-MAS), but may also, albeit rarely, complicate SLE (SLE-MAS). The clinical profile of SLE-MAS compared with SD-MAS remains unclear, as do the optimal treatment strategies.

**Methods:**

A retrospective multicentre cohort of adults with a history of SLE-MAS or SD-MAS was gathered from eight tertiary referral centres. MAS clinical/laboratory features, treatments and outcomes were compared; ‘inaugural MAS’ was defined if occurring within 6 months from the onset of the underlying disease. Exploratory multivariable profiling assessed for disease-specific features. The SLE-MAS group was also compared with large historical SLE cohorts to identify candidate risk signals.

**Results:**

While SD-MAS aligned with the prototypical hyperinflammatory pattern (more marked hyperferritinemia, very high CRP, organomegaly and liver injury; 100% meeting 2016 ACR/EULAR/PRINTO criteria), SLE-MAS (92% meeting criteria) more often featured CNS dysfunction, less liver injury, greater degree of cytopenias including anaemia, with a strong signal of autoimmune haemolysis (73% direct Coombs [DCT] positivity). Therapeutically, IL-1 antagonism was used in seven SLE-MAS with mixed results without any rapid MAS resolution. Mucocutaneous disease, autoimmune haemolysis, CNS involvement, less pronounced female preponderance and much higher DCT positivity dominated the SLE-MAS phenotype compared with historical SLE cohorts comprising 1000, 2228 and 2055 cases each.

**Conclusions:**

SLE-MAS diverged from SD-MAS with less organomegaly and liver injury but more pronounced CNS involvement and hematological involvement, the latter strongly linked to red blood cell directed autoantibodies. These findings indicate that SLE-MAS pathogenesis may be divergent and require distinct therapeutic approaches beyond extrapolation from SD-MAS.

## Graphical abstract

**Graphical Abstract:**
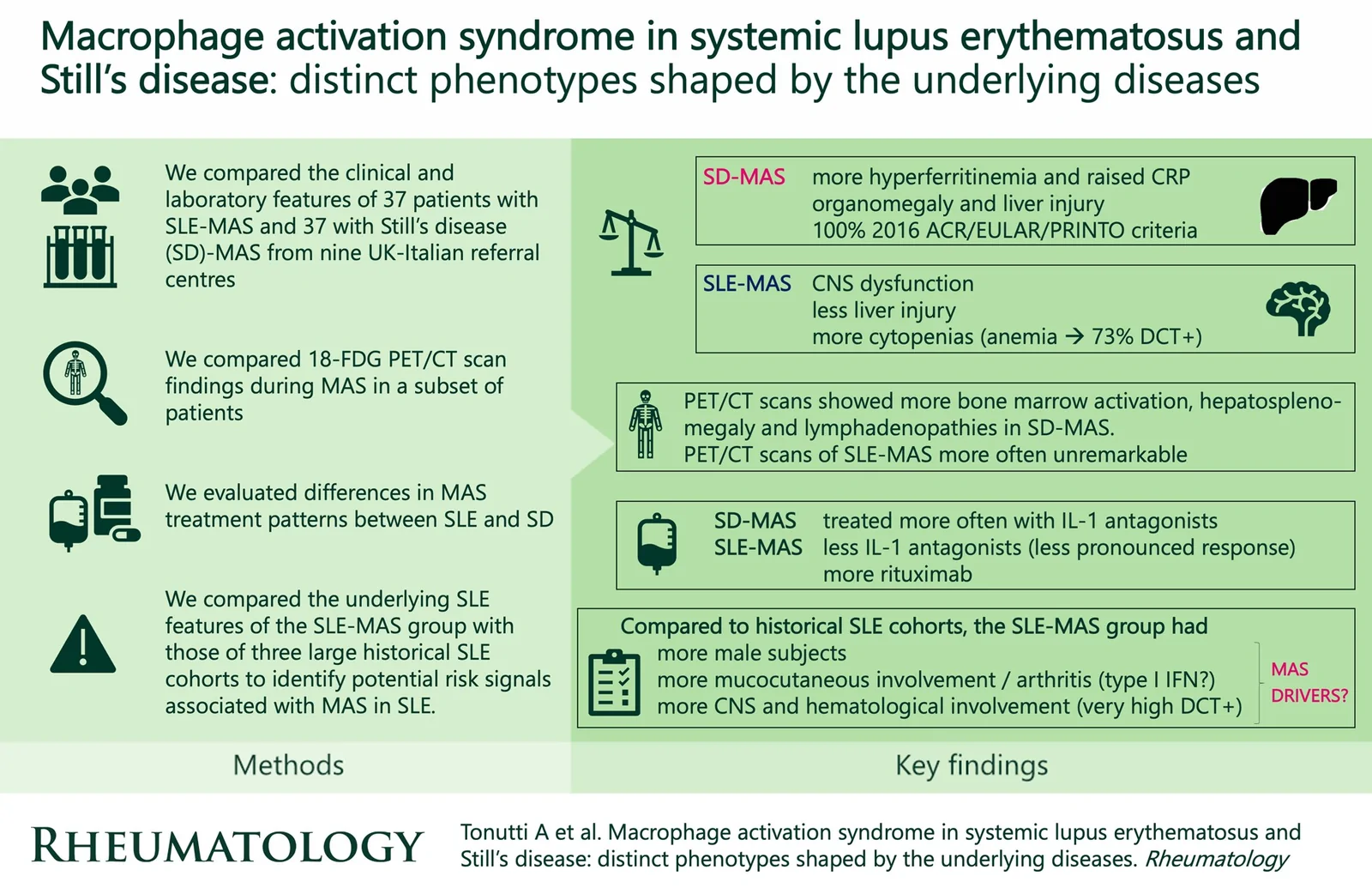

*Rheumatology* key messages

- MAS is seen in SLE, with CNS involvement and cytopenias being commoner than in Still’s disease, aligning with underlying disease predisposition.
- SLE-MAS is linked to less liver function derangement and a high rate of Coombs test positivity.
- In SLE-MAS, IL-1 antagonism with anakinra was not linked to rapid response or substantial improvements but case numbers were relatively small.

## Introduction

Macrophage activation syndrome (MAS) is a hyperinflammatory complication of malignancy, infections, inborn immune errors and autoimmune diseases [1, 2], classically associated with Still’s disease (SD), where T cell dysfunction and cytokines including IFNγ and IL-18 drive unrestrained macrophage activation linked to IL-1/IL-6-driven inflammation [3–6]. SLE is a systemic autoimmune disease with a B cell flavour and strong links to type I IFN dysregulation [7, 8]. Despite fundamental differences with SD, MAS is also recognized in SLE, but evidence remains limited to small, predominantly paediatric series, where associations with disease activity, hematological and neurological involvement are reported [9–12]. SLE-MAS characterization has been recognized as a priority among rare severe SLE manifestations [13]. Moreover, despite inadequate and unavailable evidence, the current thought that ‘MAS is MAS irrespective of the underlying disease’ is hard to challenge, and a ‘one size fits all’ approach—largely derived from SD-MAS, predominantly relying on IL-1 and calcineurin blockade [5, 6]—is endorsed for treating SLE-MAS [6, 13].

MAS is characterized by persistent fever, hyperferritinemia, hypertriglyceridaemia, cytopenias, liver injury and coagulopathy; hemophagocytes in reticuloendothelial tissues may be observed [1]. While in SD these features are captured by the 2016 EULAR/ACR/PRINTO classification criteria [14, 15] and the HScore [16, 17], it is unclear how these tools perform in SLE-MAS, where the phenotype is less delineated. Also, SLE-MAS immunopathology likely diverges from SD-MAS, since SLE-specific mechanisms—including type I IFNs and B cell activation—may predominate in driving the cytokine storm [18]. Therefore, SLE-MAS may represent a distinct subset with therapeutic implications.

The aims of this study were to: describe the features of a multicentre cohort of predominantly adult patients with SLE-MAS from international referral centres; compare SLE-MAS with SD-MAS from the same centres; summarize published adult SLE-MAS cases to assess consistency with our findings; compare the underlying SLE phenotype in our SLE-MAS cohort with three large historical SLE cohorts to identify candidate MAS risk signals.

## Methods

### Study design and setting

We performed a retrospective multicentre study including patients with SLE-MAS and SD-MAS from seven Italian and one UK tertiary referral centres. Centres collected data using a shared case report form. Regarding UK, formal ethical approval was not required as the work was a retrospective analysis conducted under the framework of an approved service evaluation of the Leeds Teaching Hospitals Trust’s Rheumatology service. Regarding Italian centres, informed written consent was obtained from all patients.

### Patient selection

Adults (≥18 years at last follow-up) were included if they fulfilled 1997 ACR or 2019 EULAR classification criteria for SLE [19, 20], or Yamaguchi’s or Fautrel’s criteria for SD [21, 22], and experienced at least one MAS episode diagnosed at the participating referral centre. Because no formal validation of the 2016 MAS classification criteria exists for SLE and adult-SD validation is limited [15], MAS was identified by expert clinician review in tertiary care settings. This approach, in line with previous series of juvenile SLE-MAS, was adopted given the absence of validated classification criteria for SLE-MAS. Adjudication relied on the presence of features supporting hyperinflammation over an isolated SLE flare, including persistent high-grade fever, haemorrhagic manifestations, hyperferritinemia and CRP elevation, and bone marrow hemophagocytosis when sampling was performed. Fulfilment of 2016 criteria [14] and HScore [16] was assessed *post hoc*.

### Clinical variables

We recorded demographics (sex, age at underlying disease onset, follow-up duration, vital status, age at the MAS episode); MAS was defined ‘inaugural’ if diagnosed within 6 months from the onset of SLE or SD. At MAS diagnosis, we collected key clinical features (fever, central nervous system—CNS involvement, organomegaly, haemorrhage, pneumonitis, myocardial damage, bone marrow hemophagocytosis) and routine laboratory parameters (blood counts, liver/kidney function, haemolysis indices, CRP, fibrinogen, triglycerides, ferritin). Remarkably, bone marrow examination was not systematically performed but at the discretion of the treating physician, as per routine practice and in line with the EULAR/ACR Points to Consider [6]. MAS treatments (glucocorticoids, immunosuppressive/immunomodulatory agents, biologics/targeted therapies, plasma exchange), intensive care unit (ICU) admissions and MAS-related mortality were recorded.

Underlying SLE phenotypes were characterized using standard definitions, including mucocutaneous, musculoskeletal, renal (proteinuria >500 mg/day or biopsy-proven nephritis [23]), CNS (delirium, psychosis, seizures, headaches) and hematological involvement (cytopenias, direct Coombs positivity), serositis, hypocomplementemia and autoantibodies [24]. SD included fever; joint involvement; skin rash; sore throat; lymphadenopathy, splenomegaly, hepatomegaly; hepatitis; serositis; pneumonitis and pulmonary fibrosis; blood count and inflammatory biomarkers.

### ^18^FDG PET/CT sub-study

A subgroup underwent ^18^F-fluorodeoxyglucose (FDG) PET/CT during MAS. Patterns of uptake were extracted from nuclear physician reports and local review, encompassing: diffuse bone marrow, liver and spleen uptake; ^18^FDG-avid lymphadenopathy; lung, joint and muscle uptake; large vessel vasculitis; malignancy/septic sources [25]. We derived a ‘diffuse uptake score’ (DUS, 0–3: one point each if diffuse bone marrow, spleen and/or liver uptake was recorded) and a ‘diffuse and nodal uptake score’ (DNUS, 0–4: DUS plus one point in case of lymphadenopathy).

### Outcomes

We compared distributions of MAS features and treatments between SLE-MAS and SD-MAS. Pre-specified sub-analyses compared: (a) patients fulfilling 2016 classification criteria [14]; (b) ICU admissions; (c) MAS-related deaths; and (d) inaugural vs non-inaugural MAS. Finally, we explored whether multivariate patterns of MAS features could distinguish SLE-MAS from SD-MAS using factor-analytic and machine-learning approaches; the aim of this analysis was purely explorative to depict any ‘disease fingerprint’ modulating MAS phenotype in SLE vs SD.

### Systematic case retrieval and comparison with historical SLE cohorts

PubMed was systematically searched on 10 December 2025 for individual-level adult case reports/series reporting SLE-MAS data using the strategy: (‘Macrophage Activation Syndrome’[Mesh] OR ‘macrophage activation syndrome’[tiab] OR MAS[tiab] OR ‘macrophage activation’[tiab] OR ‘Hemophagocytic Lymphohistiocytosis’[Mesh] OR hemophagocytic[tiab] OR hemophagocytosis[tiab] OR HLH[tiab]) AND (‘Lupus Erythematosus, Systemic’[Mesh] OR ‘systemic lupus erythematosus’[tiab] OR SLE[tiab] OR ‘lupus’[tiab]). English reports were included if missingness for variables collected in our case report form was <50%. Screening and extraction were performed by a single reviewer (A.T.) into a structured dataset.

To identify candidate risk signals associated with MAS, we compared the prevalence of SLE manifestations and autoantibodies in our SLE-MAS cohort with three multicentre historical cohorts with well-defined prevalence estimates: Euro-Lupus (*N* = 1000) [26], Hopkins Lupus (*N* = 2228) [27] and a Spanish-Portuguese binational cohort (*N* = 2055) [28]. Due to sample imbalance and lack of matching, this analysis was purely explorative.

### Statistical analysis

Continuous variables are reported as mean (±S.D.) or median (interquartile range—IQR) and compared using *t*-test or Mann–Whitney test, as appropriate; categorical variables as counts (%) and compared using *χ*^2^ or Fisher’s exact test. For continuous variables differing between the groups, receiver operating characteristic (ROC) curves were constructed and AUC, sensitivity and specificity reported.

Univariable logistic regression explored associations with ICU admission and mortality; multivariable models were not fitted due to sample size and overfitting risk. For multivariate patterning, we applied *z*-scores scaling, principal component analysis (PCA) and Uniform Manifold Approximation and Projection (UMAP) for visualization, removed zero-variance and strongly collinear variables, and tested overall group differences using PERMANOVA (Euclidean distance, 999 permutations). All clinical variables pertaining to the MAS episode were included, without a priori selection; only patients with complete data across all included variables were analysed (complete-case analysis). Supervised classification used Linear Discriminant Analysis (LDA) with 5-fold cross-validation repeated 10 times to limit overfitting given the sample size; performance was summarized by AUC-ROC, sensitivity and specificity. Standardized coefficients were extracted from the final model fitted on the full dataset for interpretability. To assess the stability, we performed a non-parametric bootstrap (1000 resamples with replacement, LDA refitted on each resample) and calculated, for each variable, the proportion of resamples in which the coefficient sign matched that of the full-dataset model (sign consistency), together with 95% bootstrap confidence intervals. All machine-learning analyses were exploratory, and estimates were interpreted cautiously.

Statistical significance was set at *P* < 0.05. FDR correction was applied across the primary comparisons presented in the main article, while it was not applied to secondary, exploratory analyses, including univariable prognostic analyses, inaugural-vs-non-inaugural comparisons, presented in the Supplementary Data [29]. Therefore, these secondary analyses should be interpreted as hypothesis-generating and are more susceptible to type I error. Analyses were performed using R version 4.4.1 (R Foundation for Statistical Computing, Vienna, Austria).

## Results

### Patients’ characteristics

We identified 37 SLE-MAS and 37 SD-MAS patients (Table 1). Among SLE patients, 23 (62%) were females; median age at SLE onset was 31 years (IQR 24–51). Mucocutaneous involvement encompassed acute 73%, subacute 35% and discoid lupus 5.4%; arthritis was present in 70%, myositis in 22%, pericarditis in 16%. Renal involvement included isolated proteinuria (35%) and biopsy-proven nephritis (24%). Neurological involvement occurred in 43%, hematological manifestations in 92%. Notably, among 26 patients with available data, direct Coombs test was positive in 73%. ANA were positive in all SLE-MAS patients, anti-dsDNA in 65%.

**Table 1:** Characteristics of patients with SLE and SD developing MAS and enrolled in the present study.

Domain	SLE clinical features (*n*/*N*, %) *N* = 37		SD clinical features (*n*/*N*, %) *N* = 37	
Mucocutaneous	Acute lupus	27 (73)	Rash	29 (78)
	Subacute lupus	13 (35)	Sore throat	22 (60)
	Discoid lupus	2 (5.4)	Fever	37 (100)
	Oral ulcers	14 (38)		
	Alopecia	9 (24)		
Musculoskeletal	Arthritis	26 (70)	Arthritis	27 (73)
	Myositis	8 (22)	Myalgia	22 (60)
Cardio-pulmonary	Pericarditis	6 (16)	Pericarditis	8 (22)
			Pleurisy	11 (30)
			Pneumonitis	6 (16)
			Lung fibrosis	3 (8.1)
Visceral	Proteinuria >500 mg/d	13 (35)	Splenomegaly	28 (76)
	Nephritis^a^	9 (24)	Hepatomegaly	23 (62)
			Hepatitis	31 (84)
Hematological	Anaemia	29 (78)	Inflammatory anaemia	30 (81)
	Direct Coombs test	19/26 (73)	Leukocytes >10.000/mmc	26 (70)
	Leukopenia	17 (46)	Leukocytes >15.000/mmc	25 (68)
	Lymphopenia	27 (73)	Neutrophilia >80%	28 (76)
	Thrombocytopenia	22 (60)	Lymphadenopathy	27 (73)
Neurological	Neuropsychiatric SLE	16 (43)		
Constitutional	Fever	34 (92)	Fever	37 (100)
	Lymphadenopathy	22 (60)	Lymphadenopathy	27 (73)
	Hepatomegaly	15 (41)	Hepatomegaly	23 (62)
	Splenomegaly	15 (41)	Splenomegaly	28 (76)
Serological	Low C3/C4	22 (60)/21 (57)	High CRP	36 (97)
	ANA	37 (100)	Hyperferritinemia	35 (95)
	Anti-dsDNA	24 (65)		
	Anti-RNP	15 (41)		
	Anti-Sm	13 (35)		
	Anti-RibP	4 (11)		
	LAC/ACLA/anti-b2GPI	9 (24)/6 (16)/7 (19)		
Treatment	Glucocorticoids	35 (95)	Glucocorticoids	37 (100)
	MTX	7 (19)	MTX	13 (35)
	CNI	11 (30)	CNI	14 (38)
	AZA	6 (16)	Anakinra	20 (54)
	CYC	4 (11)	Other IL-1 antagonists	8 (22)
	MMF	16 (43)	Tocilizumab	12 (32)
	Rituximab	15 (41)	JAK inhibitor	3 (8.1)
	Belimumab	6 (16)		
	Anifrolumab	1 (2.7)		
	HCQ	27 (73)		

Clinical features refer to cumulative, ever-occurring manifestations of the underlying disease.

a WHO classification available for eight patients: class I 1/8 (12.5); II 2/8 (25); III/V 1/8 (12.5); IV 2/8 (25); V 2/8 (25).

ACLA: anticardiolipin antibodies; b2GPI: beta-2 glycoprotein I; C3/C4: complement fractions C3 and C4; CNI: calcineurin inhibitors; LAC: lupus anticoagulant; RibP: ribosomal P.

Among SD-MAS, 22 (60%) were female, median age at SD onset was 19 years (IQR 8–41). Fever was universal, arthritis/arthralgia occurred in 73%, rash in 78%, sore throat in 60%, lymphadenopathy in 73%, splenomegaly in 76%, hepatomegaly in 62% and hepatitis in 84%. Pleurisy occurred in 30% and pericarditis in 22%. Leucocytosis was present in 70% and neutrophilia in 76%. Lung disease occurred in 16%.

### Comparison of clinical features between SLE-MAS and SD-MAS

Compared with SD-MAS, SLE-MAS patients were older at underlying disease onset (*P* = 0.025) and MAS (39 years [IQR 25–55] vs 28 years [IQR 14–43]; *P* = 0.085) (Table 2). Sex distribution and concomitant infections were comparable (38% vs 35%); these were predominantly respiratory tract infections/pneumonia, followed by bloodstream infections/endocarditis and viral infections; most were identified during hospitalization for MAS, with the exception of COVID-19, which preceded MAS onset by several months in the affected patients (Table 2). MAS was inaugural in 49% of SLE and 60% of SD cases. Median SLEDAI2K at SLE-MAS was 14 (IQR 10–23).

**Table 2:** Comparison of demographic, clinical and therapeutic features recorded in patients with SLE-MAS and SD-MAS at the index MAS episode.

	SLE (*N* = 37)	SD (*N* = 37)	*P*	Adj. *P*
Demographic				
Female sex	23 (62)	22 (60)	>0.99	>0.99
Age at SLE/SD onset	31 (24–51)	19 (8–41)	**0.004**	**0.025**
Age at MAS	39 (25–55)	28 (14–43)	**0.021**	0.085
MAS inaugural	18 (49)	22 (60)	0.484	0.752
MAS features				
Fever	36 (97)	37 (100)	>0.99	>0.99
Haemorrhage	8 (22)	5 (14)	0.541	0.787
CNS dysfunction^a^	14 (38)	6 (16)	0.067	0.204
Hepatomegaly	15 (41)	25 (68)	**0.036**	0.127
Splenomegaly	15 (41)	28 (76)	**0.005**	**0.025**
Lymphadenopathy	22 (60)	29 (78)	0.132	0.337
Pneumonitis	7 (19)	10 (27)	0.581	0.826
Myocardial damage	2 (5.4)	2 (5.4)	>0.99	>0.99
Hemophagocytosis	9 (24)	16 (43)	0.140	0.345
Infection^b^	14 (38)	13 (35)	>0.99	>0.99
WBC (cells/mm^3^)	3660 (1840–5370)	7900 (3280–15 080)	**0.001**	**0.014**
Neutrophils (cells/mm^3^)	1900 (1210–3270)	3700 (1600–8870)	**0.039**	0.131
Lymphocytes (cells/mm^3^)	570 (290–840)	1050 (550–2590)	**0.001**	**0.013**
Haemoglobin (g/dl)	9 (7.5–9.8)	9.5 (8.6–11.2)	**0.026**	0.096
Platelets (10^3^ cells/mm^3^)	100 (55–148)	100 (57–193)	0.888	>0.99
AST (IU/l)	96 (56–150)	110 (80–311)	0.065	0.204
ALT (IU/l)	75 (50–116)	143 (88–325)	**0.001**	**0.013**
ALP (IU/l)	102 (66–195)	186 (138–233)	**0.006**	**0.028**
LDH (IU/l)	547 (329–789)	785 (500–2000)	**0.017**	0.074
Creatinine (mg/dl)	0.81 (0.65–1.3)	0.75 (0.6–0.99)	0.088	0.257
CRP (mg/l)	40 (14–100)	91.8 (40.3–225.9)	**0.002**	**0.015**
PT ratio	1.1 (0.98–1.3)	1.14 (1.07–1.3)	0.151	0.348
Fibrinogen (g/l)	2.5 (1.9–3.4)	2.47 (1.3–4)	0.479	0.752
Ferritin (ng/ml)	3415 (2200–7730)	9524 (6021–24 275)	**0.001**	**0.013**
Triglycerides (mmol/l)	2.55 (1.9–4.1)	3.1 (2.39–4.2)	0.173	0.369
Sodium (mEq/l)	135 (131–140)	136 (131–139)	0.438	0.752
MAS classification criteria				
Fever	36 (97)	37 (100)	>0.99	>0.99
Ferritin >684 ng/l	37 (100)	37 (100)	>0.99	>0.99
Platelets <181.000/mm^3^	29 (78)	26 (70)	0.595	0.827
AST >48 IU/l	31 (84)	33 (89)	0.736	>0.99
Triglycerides >156 mg/dl	30 (81)	35 (95)	0.152	0.348
Fibrinogen <360 mg/dl	26 (70)	26 (70)	>0.99	>0.99
Classified as MAS	34 (92)	37 (100)	0.240	0.465
HScore	193 (160–216)	235 (204–251)	**0.001**	**0.013**
Underlying immunosuppression	0: 18 (49)	0: 23 (62)	0.350	0.659
	18: 19 (51)	18: 14 (38)		
Temperature	0: 9 (24)	0: 2 (5.4)	**0.004**	**0.025**
	33: 21 (57)	33: 16 (43)		
	49: 7 (19)	49: 19 (51)		
Organomegaly	0: 17 (46)	0: 4 (11)	**0.002**	**0.014**
	23: 12 (32)	23: 14 (38)		
	38: 8 (22)	38: 19 (51)		
Number of cytopenias	0: 1 (2.7)	0: 3 (8.1)	**0.043**	0.142
	1: 6 (16)	1: 14 (38)		
	24: 16 (43)	24: 11 (30)		
	34: 14 (38)	34: 9 (24)		
Ferritin	0: 6 (16)	0: 1 (2.7)	**0.003**	**0.020**
	35: 17 (46)	35: 8 (22)		
	50: 14 (38)	50: 28 (76)		
Triglycerides	0: 6 (16)	0: 2 (5.4)	0.159	0.351
	44: 24 (65)	44: 22 (60)		
	64: 7 (19)	64: 13 (35)		
Fibrinogen	0: 17 (46)	0: 16 (43)	>0.99	>0.99
	30: 20 (54)	30: 21 (57)		
AST	0: 4 (11)	0: 3 (8.1)	>0.99	>0.99
	19: 33 (89)	19: 34 (92)		
Hemophagocytosis	0: 27 (73)	0: 23 (62)	0.456	0.757
	35: 10 (27)	35: 14 (38)		
MAS treatment				
Steroid	34 (92)	35 (95)	>0.99	>0.99
Steroid pulses	32 (87)	32 (87)	>0.99	>0.99
Anakinra	7 (19)	20 (54)	**0.004**	**0.023**
Other IL-1 antagonist	0 (0)	3 (8.1)	0.240	0.465
Tocilizumab	0 (0)	3 (8.1)	0.240	0.465
CYC	3 (8.1)	3 (8.1)	>0.99	>0.99
IVIG	12 (32)	8 (22)	0.432	0.752
CNIs	17 (46)	21 (57)	0.485	0.752
Rituximab	12 (32)	0 (0)	**<0.001**	**0.013**
Plasma exchange	7 (19)	4 (11)	0.515	0.767
Etoposide	2 (5.4)	0 (0)	0.493	0.752
TNF inhibitor	0 (0)	1 (2.7)	>0.99	>0.99
Emapalumab	0 (0)	1 (2.7)	>0.99	>0.99
Prognosis				
Need for ICU	13 (35)	13 (35)	>0.99	>0.99
Death due to MAS	10 (27)	6 (16)	0.397	0.726
Death	13 (35)	6 (16)	0.110	0.294

ALT: alanine aminotransferase; AST: aspartate aminotransferase; CNIs: calcineurin inhibitors; CNS: central nervous system; ICU: intensive care unit; LDH: lactate dehydrogenase; WBC: white blood cells. *P*-values <0.05 are reported in **bold**.

a CNS dysfunction at MAS: seizures, CNS haemorrhage, encephalopathy, worsening status of consciousness or coma.

b Concomitant infections included respiratory tract infections/pneumonia (*n* = 9), bloodstream infections and endocarditis (*n* = 7), viral infections (*n* = 8; COVID-19, cytomegalovirus, Epstein–Barr virus, adenovirus), and isolated cases of Salmonella infection and elevated anti-streptolysin O titre (*n* = 2). Infections were identified during hospitalization for MAS, with the exception of COVID-19, which preceded MAS onset by >2 months.

SLE-MAS showed lower splenomegaly (41% vs 76%; *P* = 0.025) and hepatomegaly prevalence (41% vs 68%; *P* = 0.036), but more CNS involvement (38% vs 16%; *P* = 0.067) than SD-MAS. Laboratory profiles differed: SLE-MAS had lower WBC counts (3660/mm^3^, IQR 1840–5370, vs 7900/mm^3^, IQR 3280–15080; *P* = 0.014), deeper lymphopenia (570/mm^3^, IQR 290–840, vs 1050/mm^3^, IQR 550–2590; *P* = 0.013) and anaemia (Hb 9.0 g/dl, IQR 7.5–9.8, vs 9.5 g/dl, IQR 8.6–11.2). Markers of liver injury were lower in SLE-MAS than SD-MAS (ALT 75 IU/l, IQR 50–116, vs 143 IU/l, IQR 88–325; *P* = 0.013; ALP 102 IU/l, IQR 66–195, vs 186 IU/l, IQR 138–233; *P* = 0.028); AST did not differ substantially, consistent with ongoing haemolysis. Inflammatory markers were lower in SLE-MAS (CRP 40 mg/l, IQR 14–100, vs 92 mg/l, IQR 40–226; *P* = 0.015; ferritin 3415 ng/ml, IQR 2200–7730, vs 9524 ng/ml, IQR 6021–24275; *P* = 0.013), with similar triglycerides and fibrinogen.

Seventy-three percent of SLE-MAS patients had a positive direct Coombs test. Overall, haemolysis markers in SLE-MAS encompassed increased LDH, despite lower than SD-MAS (547 [IQR 329–789] vs 785 [IQR 500–2000] U/l), bilirubin within the normal range (0.57 [IQR 0.35–1.05] mg/dl) and a slight elevation of the reticulocyte count (3.2% [IQR 2.1–4.0]) and reticulocyte production index (1.18 [IQR 0.61–1.87]; available in *N* = 17).

The HScore was higher in SD-MAS (235, IQR 204–251) than SLE-MAS (193, IQR 160–216; *P* = 0.013), driven by fever, ferritin and organomegaly (Table 2). Overall, 92% SLE-MAS and 100% SD-MAS fulfilled the 2016 classification criteria; restricting comparisons only to these patients did not change the results.

ROC analyses showed moderate discrimination for individual biomarkers (AUC_ROC_: ferritin 0.72, WBC 0.72, ALT 0.72, ALP 0.69, CRP 0.71) and HScore (AUC_ROC_ 0.74) between SLE-MAS and SD-MAS (Supplementary Table S1).

### Treatment and outcome differences in SLE-MAS and SD-MAS

**Figure 1:**
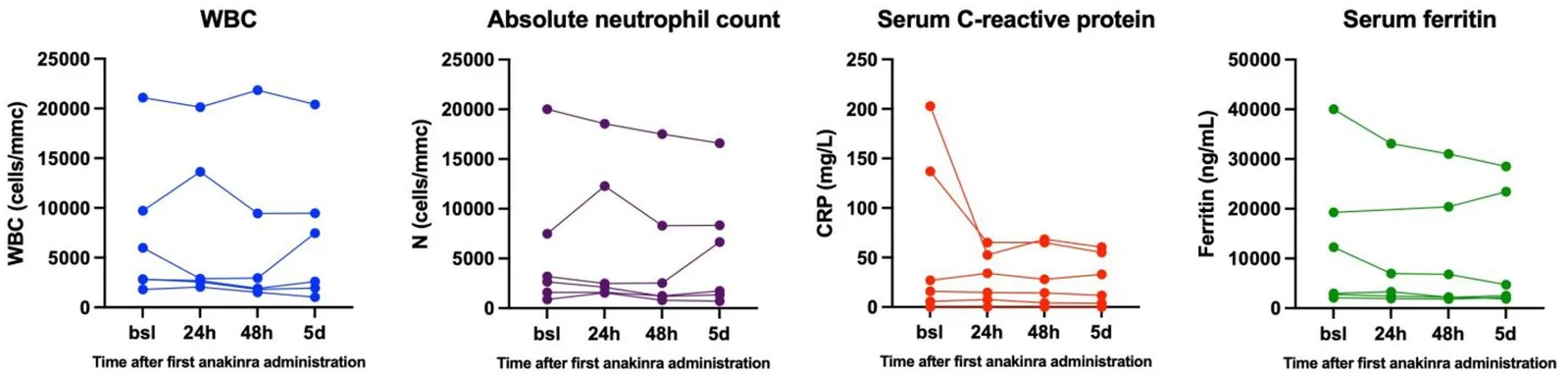
For six (of seven) SLE-MAS patients receiving anakinra, consecutively assessed inflammatory parameters were available at baseline (i.e. before the first administration), after 24 and 48 h, and after 5 days. Modest improvement in inflammatory parameters and WBC count was noted following anakinra administration. All patients were receiving concurrent glucocorticoids (0.5–1 mg/kg/d) and required additional immunosuppressive (rituximab, ciclosporin) treatment after anakinra administration

Glucocorticoids, cyclophosphamide, IVIGs, calcineurin inhibitors and plasma exchange were used at similar rates in SLE-MAS and SD-MAS. Targeted therapies diverged. IL-1 antagonists were used in 22 SD-MAS (59%): 19 patients received only anakinra, three patients only canakinumab, one patient both sequentially. Among IL-1 antagonists, only anakinra was prescribed in SLE-MAS, in a significantly lower number of patients than SD-MAS (*N* = 7, 19%; *P* = 0.023). IL-1 blockade (plus glucocorticoids but without additional immunosuppression) was adopted in 6/22 (27%) SD-MAS patients, whilst 6/7 anakinra SLE-MAS cases required additional interventions (Supplementary Table S2). Anakinra was associated with a modest and slow decline in inflammatory parameters in the seven SLE-MAS patients receiving it (Fig. 1); given the small, non-randomized subgroup and heterogeneous concomitant therapies, this observation is purely descriptive. Rituximab was used only in SLE-MAS (32%; *P* = 0.013); tocilizumab (*N* = 3), etanercept and emapalumab (*N* = 1 each) only in SD-MAS.

ICU admission occurred in 35% of both the groups; MAS-related (27% vs 16%) and overall mortality (35% vs 16%) were numerically higher in SLE-MAS despite not reaching significance. In SD, all deaths were MAS-related; in SLE, 3/13 deaths were seen during follow-up, related to underlying disease complications or comorbidities.

### Need for ICU and mortality in SLE-MAS and SD-MAS

Thirteen patients per group were admitted to ICU (Supplementary Table S3). Among these, CNS dysfunction was more common in SLE-MAS (85%) than SD-MAS (31%; *P* = 0.015). ICU-admitted SLE-MAS patients also had lower lymphocyte counts, CRP and HScore (205, IQR 154–223, vs 243, IQR 236–254; *P* = 0.031) than the SD-MAS counterpart (Supplementary Table S3). Among patients succumbing to MAS (10 SLE; 6 SD), clinical/demographic features did not differ, acknowledging limited sample size.

In univariable models (Supplementary Table S4), ICU admission and death in SLE-MAS were associated with CNS dysfunction, pneumonitis, concomitant infection and lower platelet count. In SD-MAS, ICU admission and death were associated with older age, haemorrhagic diathesis, pneumonitis, increased creatinine and concomitant infection. Regarding treatments, higher odds of ICU admission were observed with anakinra and plasma exchange in SLE-MAS, while no significant association was reported for SD-MAS; these associations may reflect preferential use of these therapies in more severe cases rather than a detrimental treatment effect.

### Milder phenotype in ‘inaugural’ SD-MAS but not SLE-MAS

Overall, inaugural MAS cases were younger (*P* = 0.008), had fewer documented infections (22.5% vs 53%; *P* = 0.014) and lower mortality (10% vs 35%; *P* = 0.011) compared with non-inaugural cases (Supplementary Table S5).

In SLE-MAS, inaugural vs non-inaugural events showed no clear differences, but direct Coombs positivity was higher in inaugural cases (93% vs 50%; *P* = 0.026). In SD-MAS, inaugural cases were younger (*P* = 0.013), with fewer ICU admissions (18% vs 60%; *P* = 0.015), deaths (4.5% vs 33%; *P* = 0.031) and infections (18% vs 60%; *P* = 0.015). Inaugural SD-MAS showed higher WBC (*P* = 0.015) and platelets (*P* = 0.020), and lower HScore (*P* = 0.049). Compared with non-inaugural SD-MAS, these patients were characterized by more skin rash, less pleurisy and pneumonitis (Supplementary Table S6).

### Machine learning reveals distinct patterns of MAS features in SLE vs SD

**Figure 2:**
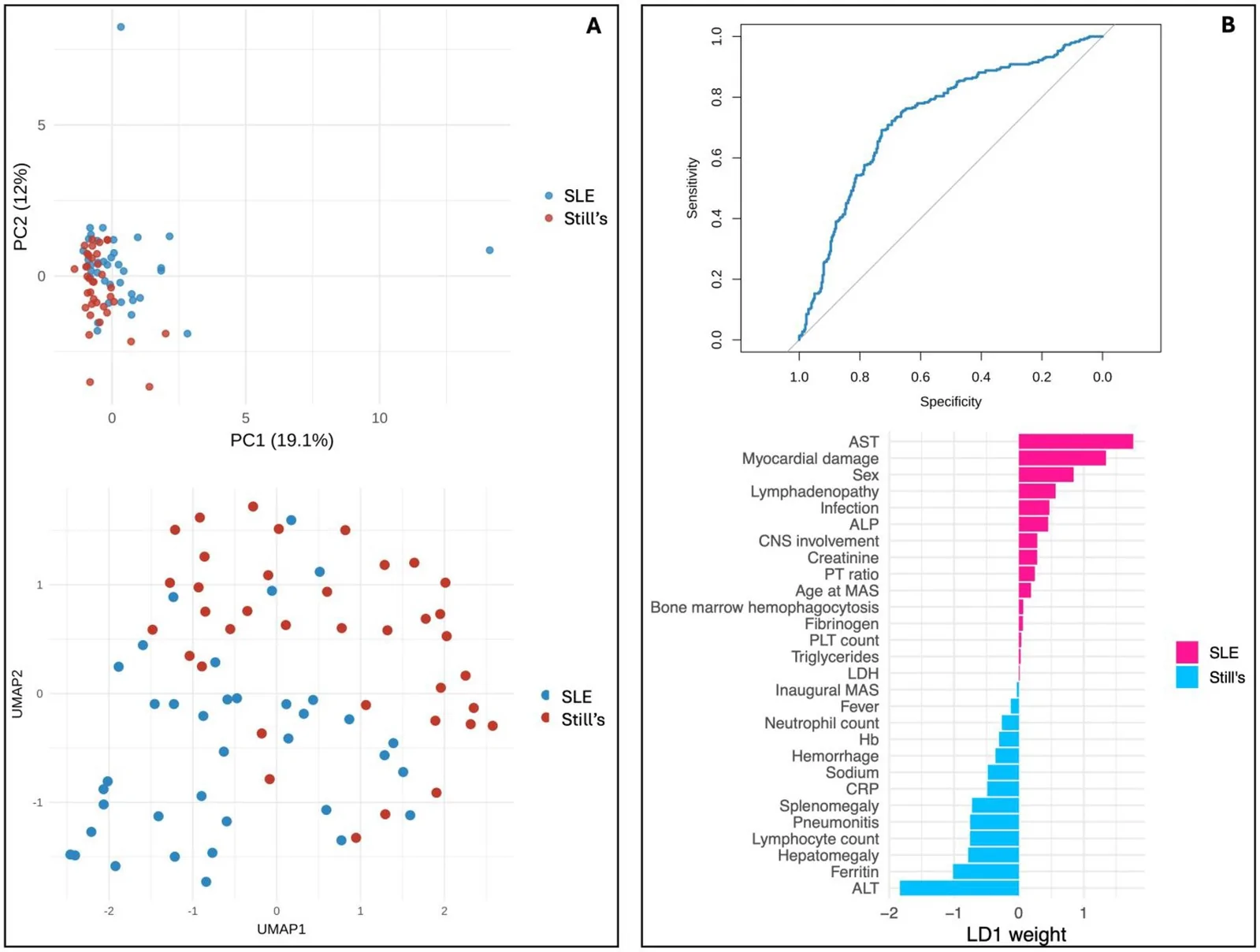
Supervised and unsupervised machine-learning analysis of MAS features reveal distinct patterns in SLE and SD. (**A**) PCA and UMAP revealed only minor separation, despite significance was reached on PERMANOVA. (**B**) LDA with 5-fold cross-validation showed moderate accuracy (AUC-ROC 0.73) in distinguishing SLE and SD, the weight of different features reported in the bar plot

Exploratively, unsupervised dimensionality reduction (PCA/UMAP) suggested partial overlap with modest separation (Fig. 2). PERMANOVA demonstrated a small but statistically significant difference in multivariate MAS profiles (*R*^2^ = 0.05; *P* = 0.001), indicating that, while distinguishable as groups, SLE-MAS and SD-MAS profiles may overlap at the individual-patient level. LDA achieved moderate cross-validated discrimination (AUC-ROC 0.73; sensitivity 0.71; specificity 0.70; accuracy 0.70), confirmed on bootstrap stability analysis (Supplementary Fig. S1, Supplementary Table S7).

### ^18^FDG PET/CT features in SLE-MAS and SD-MAS

**Figure 3:**
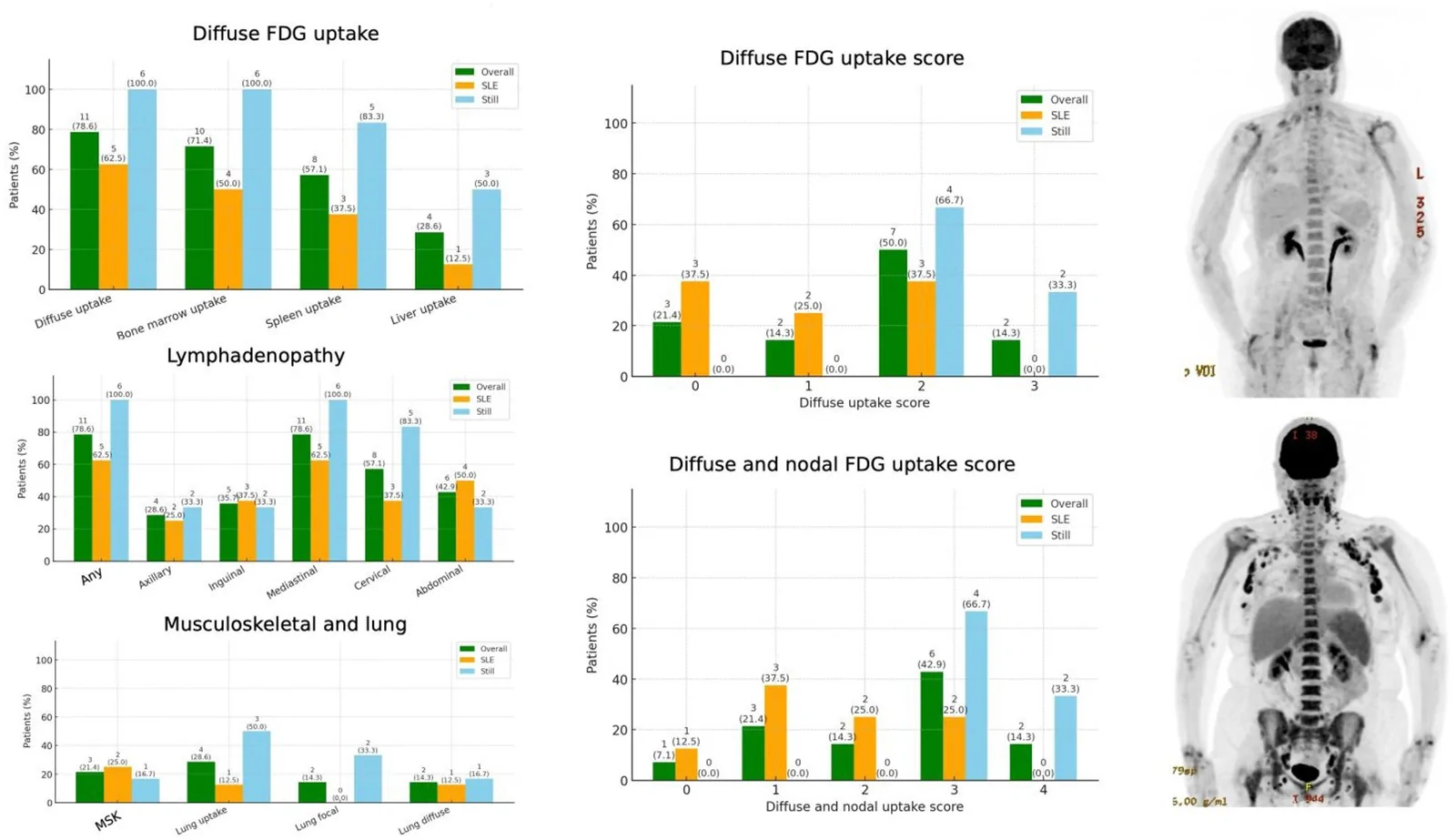
Comparison of ^18^FDG uptake patterns on PET/CT scans in patients with SLE-MAS (*N* = 8, orange) and SD-MAS (*N* = 6, sky blue). Median ‘diffuse uptake score’ and ‘diffuse and nodal uptake score’ were significantly higher in SD-MAS compared with SLE-MAS. In the right quadrant, two examples of ^18^FDG PET/CT scans highlighting capsulitis/arthritis and modest lymphadenopathy in a SLE patient (top), and florid and diffuse ^18^FDG avid lymphadenopathies, diffuse liver, spleen and bone marrow uptake in a patient with Still’s disease (bottom)

^18^FDG PET/CT performed during MAS was available for eight SLE-MAS and six SD-MAS patients (Fig. 3). Diffuse marrow, liver and spleen uptake tended to be more common in SD-MAS, and the DUS was higher in this group (median 2, IQR 2–3) than SLE-MAS (median 1, IQR 0–2; *P* = 0.01). DNUS was also higher in SD-MAS (3, IQR 3–4, vs 2, IQR 2–3; *P* = 0.007). Pulmonary uptake was more frequent in SD-MAS (3/6) than SLE-MAS (1/8), with focal consolidations in two SD cases. Arthritis/capsulitis was observed in two SLE-MAS and one SD-MAS. Due to the limited size of the unselected sample undergoing PET/CT, these results are purely exploratory.

### Systematic retrieval of published SLE-MAS cases

The search yielded 654 PubMed records: 96 articles reporting 102 individual SLE-MAS cases were included (Supplementary Table S8). The phenotype was broadly concordant with our cohort: predominant hematological involvement and severe systemic manifestations (Supplementary Table S9). Potential under-reporting could have affected neurological features (23%) and organomegaly (often with unclear clinical/imaging definitions). Direct Coombs positivity remained prominent (58%), supporting autoimmune haemolysis as a recurrent hallmark. Treatment patterns mirrored conventional approaches, rituximab and anakinra were reported in small subgroups; one isolated report of type I IFN blockade (anifrolumab) showed efficacy [30].

### Explorative comparison with historical cohorts reveals putative risk factors for MAS in SLE

The demographics and phenotype of SLE-MAS patients were compared with three historical SLE cohorts to describe putative signals associated with MAS in SLE (Table 3). Male sex was more frequent in SLE-MAS (38% vs 8–12.5%; *P* < 0.005), where mucocutaneous and articular manifestations were also commoner. Neurological involvement (43% vs 9–19%; *P* < 0.002), thrombocytopenia and direct Coombs positivity (73% vs 14–21%; *P* < 0.003) were markedly enriched in SLE-MAS.

**Table 3:** Comparison of demographic and clinical features of SLE recorded in our SLE-MAS cohort and in three historical SLE cohorts, namely the Euro-Lupus project [26], the Hopkins Lupus cohort [27] and the Spanish-Portuguese cohort [28].

	SLE-MAS (*N* = 37)	Euro-Lupus (*N* = 1000)	*P*	Adj. P	Hopkins Lupus (*N* = 2228)	*P*	Adj. *P*	Spanish Portuguese (*N* = 2055)	*P*	Adj. *P*
Female sex	23 (62)	875 (88)	**<0.001**	**0.002**	2054 (92)	**<0.001**	**0.003**	1878 (91)	**<0.001**	**<0.001**
Age at SLE onset	37 (18)				32 (13)	0.106	0.163	33 (14)	0.162	0.364
Acute cutaneous lupus	27 (73)	311 (31)	**<0.001**	**0.002**	1090/2226 (49)	**0.004**	**0.010**	1385 (67)	0.473	0.735
Subacute cutaneous lupus	13 (35)	67 (6.7)	**<0.001**	**0.002**	118/2224 (5.3)	**<0.001**	**0.003**			
Discoid lupus	2 (5.4)	78 (7.8)	>0.99	>0.99	444/2224 (20)	**0.027**	0.056	212 (10)	0.579	0.735
Oral ulcers	14 (38)	125 (13)	**<0.001**	**0.002**	1181/2226 (53)	0.066	0.117	727 (35)	0.756	0.808
Alopecia	9 (24)				1266/2225 (57)	**<0.001**	**0.003**	592 (29)	0.550	0.735
Arthritis	26 (70)	481 (48)	**0.008**	**0.012**	1607/2220 (72)	0.775	0.841	1490 (73)	0.763	0.808
Myositis	8 (22)	43 (4.3)	**<0.001**	**0.002**	178/2219 (8.0)	**0.009**	**0.021**			
Proteinuria	13 (35)				1025/2221 (46)	0.182	0.246			
Nephritis	9 (24)	279 (28)	0.633	0.748				604 (29)	0.502	0.735
Asymptomatic effusions	13 (35)				1101/2225 (50)	0.083	0.136			
Pericarditis	6 (16)	160 (16)	0.972	>0.99				473 (23)	0.329	0.592
Fever	34 (92)	166 (17)	**<0.001**	**0.002**						
Neurologic involvement	16 (43)	194 (19)	**<0.001**	**0.002**				177 (8.6)	**<0.001**	**<0.001**
Raynaud’s	9 (24)	163 (16)	0.198	0.257	1156/2224 (52)	**<0.001**	**0.003**			
Hematological SLE	34 (92)				1391/2057 (68)	**0.002**	**0.006**	1377 (67)	**0.001**	**0.004**
Anaemia	29 (78)							228 (11)	**<0.001**	**<0.001**
Direct Coombs test	19/26 (73)				404/1911 (21)	**<0.001**	**0.003**	182/1309 (14)	**<0.001**	**<0.001**
Leukopenia	17 (46)				1066/2224 (48)	0.810	0.841	859 (42)	0.612	0.735
Thrombocytopenia	22 (60)	134 (13)	**<0.001**	**0.002**	467/2223 (21)	**<0.001**	**0.003**	395 (19)	**<0.001**	**<0.001**
Low complement C3	22 (60)				1255/2226 (56)	0.708	0.814			
Low complement C4	21 (57)				1075/2226 (48)	0.307	0.392			
Antinuclear antibodies	37 (100)							2032 (99)	>0.99	>0.99
Anti-dsDNA	24 (65)				1407/2224 (63)	0.841	0.841	1527 (74)	0.194	0.387
Anti-RNP	15 (41)				644/2177 (30)	0.148	0.213			
Anti-Sm	13 (35)				470/2183 (22)	**0.047**	0.090	312 (15)	**<0.001**	**0.003**
Lupus anticoagulant	9 (24)				590/2178 (27)	0.707	0.814			
Anticardiolipin antibodies	6 (16)				1063/2176 (49)	**<0.001**	**0.003**			

Empty cells are left in case data were not available in the original manuscript for selected manifestations. *P*-values <0.05 are reported in **bold**.

## Discussion

In this multicentre cohort—one of the largest predominantly adult studies comparing SLE-MAS and SD-MAS—both conditions fulfilled a recognizable MAS framework, yet their phenotype differed in clinically relevant ways. These divergences, not always obvious at first glance, became evident when examining the relative ‘weight’ of organ involvement and laboratory alterations, and tracked closely with the underlying diseases, supporting the concept that MAS is a shared final common pathway whose clinical–biological signature might be induced and modulated by different disease-specific immune programs. These divergent clinical phenotypes may be compatible with previously reported differences in serum cytokines between SLE-MAS, showing a more prominent type I IFN signature, vs SD-MAS, dominated by IL-1/IL-18 [31]. Of note, this remains a speculative interpretive framework, and the correlation between serum cytokines and the phenotypes observed in our cohort should be explored also therapeutically.

Across demographics and MAS features, SLE-MAS was characterized by older age, higher frequency of CNS dysfunction and deeper cytopenias—leukopenia and anaemia—than SD-MAS, extending previous observations [32]. Nonetheless, the age difference between the SLE-MAS and SD-MAS should be accounted as a potential confounder for some of the phenotypic and prognostic differences described, requiring more exhaustive evaluation in larger cohorts. The high prevalence of direct Coombs positivity in SLE-MAS (73%) points to an association warranting dedicated mechanistic investigation, despite our data were not powered to directly support overt ongoing autoimmune haemolysis as a feature of SLE-MAS, in keeping with the multifactorial genesis of anaemia in MAS and the bone marrow suppression associated with the cytokine storm. Whether the binding of antibody-coated erythrocytes to reticuloendothelial macrophages contributes to a distinct pattern of erythrophagocytosis in SLE-MAS remains speculative and requires dedicated investigation. Inflammatory markers provided an additional discriminant, with CRP and ferritin being significantly lower in SLE-MAS than SD-MAS. This highlights a diagnostic pitfall since SLE-MAS may present without the florid ‘hyperinflammatory/organomegaly/liver injury’ constellation characteristic of SD-MAS [3], despite retaining similar severity requiring early recognition.

The performance of available MAS tools was coherent with the described phenotypes. The 2016 classification criteria, designed for juvenile SD and subsequently applied to adult-SD [6, 14], captured essentially all SD-MAS and most SLE-MAS cases in our cohort, but SLE-MAS met some items less consistently. Likewise, HScore values were lower in SLE-MAS, driven by differences in temperature, ferritin and organomegaly [16]. While our study is not powered to validate these tools in SLE, our findings emphasize that meeting criteria does not equate to identical biology across diseases: indeed, critical involvement characterized by CNS dysfunction [33] and profound cytopenias may dominate in SLE-MAS but be not adequately captured by these tools because of the less extreme hyperinflammatory signal. Despite their explorative nature, machine-learning analyses supported the same conceptual model, revealing substantial overlap but still detectable SLE vs SD ‘fingerprints’ in MAS phenotype.

Prognosis was comparable between the groups, with a numerical trend towards higher mortality in SLE-MAS, aligning with paediatric series [9, 34]. Prognostic signals overlapped across diseases—older age, pneumonitis, kidney injury and infection—supporting these as cross-cutting drivers of severe MAS, in line with a recent European population-based study including all MAS cases independently from the aetiology [2, 5]. However, thrombocytopenia [5, 12, 35] and CNS dysfunction emerged as markers of ICU admission and death in SLE-MAS [6]. The inaugural-vs-non-inaugural analysis further underscored disease-specific dynamics: inaugural SD-MAS tended to be milder, with fewer ICU admissions and deaths and less visceral involvement, whereas SLE-MAS did not show the same ‘attenuation’ when inaugural. To what extent such differences relate to demographic confounders (younger age in SD-MAS) or disease-related alterations requires further research.

IL-1 blockade was used more frequently in SD-MAS (*N* = 22) than in SLE-MAS (*N* = 7), and a substantial proportion of SD-MAS patients could be managed without additional immunosuppression beyond glucocorticoids. In contrast, SLE-MAS more often required adjunctive disease-modifying strategies (*N* = 6), including rituximab [10, 34]. We observed modest and relatively slow changes in serum CRP and ferritin following anakinra in SLE-MAS, in apparent contrast to the dramatic responses reported in SD-MAS [36]. Given the small size of this subgroup (*N* = 7), the non-randomized allocation of anakinra, and the heterogeneity of concomitant therapies, this observation may be cautiously seen as a signal of reduced efficacy of IL-1 blockade in SLE-MAS. While in SD-MAS IL-1 inhibition remains strongly supported by the pathogenic continuum of SD [6, 37–39], its role in SLE-MAS is less defined [37] and alternative approaches, such as B cell depletion or type I IFN blockade [40, 41], have shown encouraging results [30]. Future prospective studies should test whether a cytokine-driven treatment of MAS can offer additional benefit over current paradigms.

Finally, the comparison with historical SLE cohorts provides plausible candidate risk signals that are recognizable and partially align with previous paediatric studies [9, 10, 34] and small series [11, 42]. These include male sex, mucocutaneous lupus, CNS and hematological involvement, with a notably very high prevalence of direct Coombs positivity. Rather than implying causality, these signals support a model in which SLE-specific immune programs—potentially including autoantibody-mediated cytopenias, reticuloendothelial activation [10, 42] and IFN-driven inflammation—shape MAS phenotype. These hypotheses can inform future prospective risk-factor analyses.

This study has limitations. Its retrospective design and setting may introduce selection and referral bias, potentially enriching for more complex cases, limiting generalizability to unselected SLE and SD populations. Mirroring real-world practice, MAS was defined by expert clinician diagnosis rather than relying on classification criteria, which are not validated for SLE and were assessed *post hoc* along with the HScore. We cannot exclude that some episodes adjudicated as MAS could represent severe SLE flares, an inherent limitation of retrospective cohorts. The overlap between CNS manifestations of MAS *vs* neuropsychiatric SLE should be also accounted, while these are uncommon in uncomplicated adult-SD. This is however applicable also to SD-MAS, since severe SD flares are seldom difficult to differentiate from cases complicated by MAS, the treatment remaining similar—if not the same—in both scenarios. Also, features supporting the distinction between SLE-MAS and severe SLE flare were reported in most cases, including high-grade fever, haemorrhagic manifestations, hyperferritinemia, CRP elevation and bone marrow hemophagocytosis. In addition, while individual SLE manifestations at the precise time of MAS could not be reliably disentangled from the acute clinical presentation and SLEDAI was retrospectively reconstructed from clinical records, SLEDAI scores at the time of MAS were not uniformly elevated to a degree that would, by itself, account for a severe SLE flare.

Centre-level heterogeneity in diagnostic work-up and treatment strategies are inherent to multicentre retrospective studies, although we mitigated this through a shared case report form focusing on coreset variables which thoroughly reduced the risk of missingness. However, bone marrow examination was performed at the discretion of the treating physician rather than systematically, reflecting real-world practice and EULAR/ACR points to consider. Notably, the absence of bone marrow haemophagocytosis should not exclude the diagnosis of MAS [6]. Haemolysis parameters, including haptoglobin and transfusion requirements, were not systematically available and further studies should investigate on the possible contribution of DCT positivity/autoimmune haemolysis as a driver of SLE-MAS. ^18^FDG PET/CT was available only in a small patient subset with unclear selection criteria, and the findings of this sub-study should be interpreted as explorative.

The clinical significance and contribution to MAS of infectious findings remained uncertain, and no patient was adjudicated as having infection-associated HLH independent of the underlying rheumatic disease. Nonetheless, we cannot exclude that infection contributed, at least in part, as a concomitant trigger or amplifying factor in some cases. Given sample size constraints and the difficulty in unambiguously classifying infection-associated status, a sensitivity analysis excluding all infection-associated cases was not reliably performed. It is worth noting that infection related to immunodeficiency status—reminiscent of primary HLH—can occur in SD, and SLE is *per se* associated with immunodeficiency including complement deficiency and impaired T and B cell activation [43]. Also, the immunosuppressive regimens used to treat SLE-MAS and SD-MAS should be accounted, and a careful infectious screening may be warranted in cases complicated by MAS.

Despite being among the largest adult series to date, sample size remains limited for robust modelling; regression analyses were restricted to univariable associations and should be interpreted as purely hypothesis-generating. Machine-learning analyses were explicitly exploratory, despite bootstrap resampling suggested stable and consistent results even in a small dataset. The explored treatment––outcome associations are subject to confounding by indication given non-randomized treatment allocation; thus, the findings are explorative, should not be interpreted as evidence of comparative efficacy, and should be regarded with caution.

In conclusion, SLE-MAS and SD-MAS exhibit phenotypic–therapeutic divergences that track with the underlying diseases. SD-MAS more often conforms to the prototypical hyperinflammatory profile (hyperferritinemia, increased CRP, organomegaly, liver injury) whereas SLE-MAS is frequently dominated by CNS dysfunction and cytopenias, with a prominent signal of autoimmune haemolysis—something relevant when supervening on the intrinsic MAS hemophagocytes. Recognizing these differences should heighten early suspicion of SLE-MAS when classical hyperinflammatory cues are muted and, crucially, argues against a ‘one-size-fits-all’ therapeutic extrapolation from SD-MAS. While IL-1 blockade is biologically-anchored and effective in SD-MAS, SLE-MAS may benefit from strategies more specifically tailored to SLE immunopathology, such as B cell- or type I IFN-directed approaches, something worth testing in prospective studies. Our findings nonetheless support the broader principle that MAS diagnosis and management may benefit from consideration of the underlying disease rather than a uniform approach.

## Supplementary Material

keag490_Supplementary_Data

## Data Availability

The data underlying this article will be shared upon reasonable request to the corresponding author.
