# Supplementary material for: Macrophage activation syndrome in systemic lupus erythematous and Still’s disease: distinct phenotypes shaped by the underlying diseases
Source: Rheumatology (Oxford). 2026 Sep 11;65(9):keag490. doi: 10.1093/rheumatology/keag490 (PMC13612172; doi:10.1093/rheumatology/keag490)
Supplement: Supplementary file 1 [file keag490_supplementary_data.docx]

**Supplementary Table S1**. Receiver operating characteristic (ROC)-derived thresholds for selected biomarkers in SLE-MAS vs SD-MAS.

| **Variable** | **AUC-ROC** | **Threshold** | **Sensitivity** | **Specificity** |
| --- | --- | --- | --- | --- |
| Ferritin (ng/mL) | 0.72 | 6009 | 0.65 | 0.76 |
| WBC (cells/mm^3^) | 0.72 | 8080 | 0.95 | 0.49 |
|  |  | 6990 | 0.89 | 0.54 |
| ALT (IU/L) | 0.72 | 117 | 0.76 | 0.65 |
|  |  | 87 | 0.59 | 0.81 |
| ALP (IU/L) | 0.69 | 136 | 0.62 | 0.76 |
|  |  | 87 | 0.46 | 0.92 |
|  |  | 81 | 0.43 | 0.95 |
| CRP (mg/L) | 0.71 | 35.7 | 0.49 | 0.89 |
| HScore | 0.74 | 236 | 0.81 | 0.62 |
|  |  | 219 | 0.73 | 0.70 |
|  |  | 206 | 0.65 | 0.78 |
|  |  | 204 | 0.62 | 0.81 |

**Supplementary Table S2**. Comparison between SLE-MAS and SD-MAS patients receiving anakinra or other IL-1 antagonists for MAS.

| **Variable** | **SLE-MAS anakinra**  **(N=7)** | **SD-MAS anakinra**  **(N=22)** | **p** |
| --- | --- | --- | --- |
| **Demographic features** |  |  |  |
| Age at disease onset | 27 (25–47) | 22 (9.5–43) | 0.185 |
| Age at MAS | 30 (26–55) | 30 (15–49) | 0.320 |
| Sex | 2 (28.6) | 13 (59.1) | 0.215 |
| Follow-up duration | 1 (0.5–7) | 5 (1–12) | 0.523 |
| Infection | 4 (57.1) | 10 (45.5) | 0.682 |
| Inaugural MAS | 6 (85.7) | 13 (59.1) | 0.367 |
| Need for ICU | 6 (85.7) | 10 (45.5) | 0.093 |
| Death due to MAS | 3 (42.9) | 5 (22.7) | 0.357 |
| **MAS phenotype** |  |  |  |
| Fever | 7 (100) | 22 (100) | >0.99 |
| Hemorrhage | 2 (28.6) | 3 (13.6) | 0.569 |
| CNS dysfunction | 5 (71.4) | 5 (22.7) | **0.030** |
| Hepatomegaly | 3 (42.9) | 14 (63.6) | 0.403 |
| Splenomegaly | 2 (28.6) | 17 (77.3) | **0.030** |
| Lymphadenopathy | 4 (57.1) | 18 (81.8) | 0.311 |
| Pneumonitis | 3 (42.9) | 7 (31.8) | 0.665 |
| Hemophagocytosis | 3 (42.9) | 10 (45.5) | >0.99 |
| **MAS chemistries** |  |  |  |
| WBC (cells/mm3) | 2840 (2550–5675) | 6325 (4078–13028) | 0.154 |
| ANC (cells/mm3) | 2650 (1915–3100) | 3400 (1790–7765) | 0.400 |
| ALC (cells/mm3) | 570 (245–775) | 955 (570–2370) | 0.063 |
| Hb (g/dL) | 9 (7.3–9.1) | 9.6 (8.2–11.4) | 0.097 |
| PLT (x103 cells/mm3) | 47 (34.5–77.5) | 117 (59.5–213) | **0.037** |
| AST (IU/L) | 96 (75–159) | 109 (71–311) | 0.524 |
| ALT (IU/L) | 60 (39–114) | 152 (88–320) | 0.056 |
| ALP (IU/L) | 102 (78–161) | 171 (128–209) | 0.161 |
| LDH (IU/L) | 789 (596–805) | 793 (463–2052) | 0.610 |
| Creatinine (mg/dL) | 1.4 (0.9–1.8) | 0.82 (0.61–1.15) | 0.056 |
| CRP (mg/L) | 16 (6.3–84.5) | 131.5 (51.5–216.7) | **0.016** |
| Fibrinogen (g/dL) | 2.42 (2.1–3) | 2.5 (1.4–3.4) | 0.919 |
| Ferritin (ng/L) | 12286 (2881–22391) | 11535 (5363–29673) | 0.646 |
| Triglycerides (mmol/L) | 3.0 (2.8–5) | 2.6 (2.3–3.9) | 0.593 |
| **H-Score** | 200 (177–213) | 235 (214–244) | **0.005** |
| **Classified as MAS** | 7 (100) | 22 (100) | >0.99 |
| **MAS treatment** |  |  |  |
| Glucocorticoids | 7 (100) | 21 (95.5) | >0.99 |
| Tocilizumab | 0 (0) | 3 (13.6) | 0.558 |
| Cyclophosphamide | 2 (28.6) | 2 (9.1) | 0.238 |
| IVIG | 3 (42.9) | 7 (31.8) | 0.665 |
| CNI | 4 (57.1) | 13 (59.1) | >0.99 |
| Rituximab | 2 (28.6) | 0 (0) | **0.052** |
| Plasma exchange | 3 (42.9) | 3 (13.6) | 0.132 |
| Etoposide | 2 (28.6) | 0 (0) | **0.052** |
| Anti-TNF | 0 (0) | 0 (0) | >0.99 |
| Emapalumab | 0 (0) | 1 (4.5) | >0.99 |

*ALP: alkaline phosphatase; ALT: alanine aminotransferase; AST: aspartate aminotransferase; CNIs: calcineurin inhibitors; CNS: central nervous system; CRP: C-reactive protein; ICU: intensive care unit; IVIG: intravenous immunoglobulins; LDH: lactate dehydrogenase; WBC: white blood cells.*

**Supplementary Table S3.** Sub-analysis: comparison of patients with SLE-MAS and SD-MAS admitted to ICU and dying of MAS.

|  | **Patients admitted to ICU** | | | **Patients dying of MAS** | | |
| --- | --- | --- | --- | --- | --- | --- |
| **Variable** | **SD-MAS**  **(N = 13)** | **SLE-MAS**  **(N = 13)** | **p** | **SD-MAS**  **(N = 6)** | **SLE-MAS**  **(N = 10)** | **p** |
| **Demographic features** |  |  |  |  |  |  |
| Sex | 6 (46) | 6 (46) | >0.99 | 2 (33) | 3 (30) | >0.99 |
| Age at disease onset | 25 [15-53] | 28 [25-53] | 0.396 | 38 [17-61] | 58 [30-65] | 0.231 |
| Age at MAS | 43 [28-51] | 39 [27-55] | 0.898 | 51 [45-68] | 59 [49-71] | 0.625 |
| MAS inaugural | 4 (31) | 7 (54) | 0.428 | 1 (17) | 3 (30) | >0.99 |
| **MAS phenotype** |  |  |  |  |  |  |
| Hemorrhage | 4 (31) | 4 (31) | >0.99 | 4 (67) | 4 (40) | 0.608 |
| CNS dysfunction | 4 (31) | 11 (85) | **0.015** | 4 (67) | 8 (80) | 0.604 |
| Hepatomegaly | 8 (62) | 6 (46) | 0.695 | 4 (67) | 4 (40) | 0.608 |
| Splenomegaly | 9 (69) | 6 (46) | 0.428 | 6 (100) | 5 (50) | 0.093 |
| Lymphadenopathy | 10 (77) | 7 (54) | 0.411 | 6 (100) | 6 (60) | 0.234 |
| Pneumonitis | 8 (62) | 6 (46) | 0.695 | 4 (67) | 5 (50) | 0.633 |
| Myocardial damage | 1 (7.7) | 2 (15) | >0.99 | 0 (0) | 0 (0) | - |
| Hemophagocytosis | 8 (62) | 4 (31) | 0.238 | 5 (83) | 2 (20) | **0.035** |
| Infection | 8 (62) | 9 (69) | >0.99 | 5 (83) | 7 (70) | >0.99 |
| **MAS chemistries** |  |  |  |  |  |  |
| WBC (cells/mm^3^) | 5400 [2700-12050] | 2840 [1940-5980] | 0.293 | 2575 [890-3863] | 3515 [2130-6495] | 0.428 |
| Neutrophils (cells/mm^3^) | 2358 [420-8070] | 2650 [1340-4560] | 0.96 | 1979 [528-2787] | 2815 [1575-5797.5] | 0.313 |
| Lymphocytes (cells/mm^3^) | 1100 [240-4200] | 310 [230-420] | **0.057** | 225 [158-435] | 340 [267.5-645] | 0.428 |
| Hemoglobin (g/dL) | 8.7 [7.7-9.8] | 8.6 [7.5-9.1] | 0.258 | 8.35 [7.78-9.67] | 9 [7.78-9.1] | 0.913 |
| Platelets (cells/mm^3^) | 57 [14-220] | 55 [39-89] | 0.739 | 28 [11-54] | 46 [33.25-76] | 0.447 |
| AST (IU/L) | 108 [85-311] | 96 [55-199] | 0.355 | 63 [32-104] | 100 [54.25-182] | 0.587 |
| ALT (IU/L) | 145 [70-305] | 62 [31-157] | 0.085 | 57 [25-89] | 61 [25-164.5] | >0.99 |
| ALP (IU/L) | 202 [145-378] | 111 [76-417] | 0.124 | 197 [190-334] | 272 [90.75-444] | 0.713 |
| LDH (IU/L) | 1058 [591-2149] | 690 [440-805] | 0.081 | 1188 [602-3484] | 796.5 [567-906.25] | 0.492 |
| Creatinine (mg/dL) | 1.08 [0.9-1.2] | 1.13 [0.8-1.93] | 0.412 | 1.15 [1.01-1.49] | 1.44 [1.07-1.9] | 0.428 |
| CRP (mg/L) | 170 [59-226] | 32 [7-146] | **0.058** | 198 [110-241] | 91 [32-140] | 0.093 |
| PT ratio | 1.2 [1.14-1.32] | 1.2 [0.98-1.4] | 0.456 | 1.23 [1.14-1.32] | 1.25 [1.19-1.32] | >0.99 |
| Fibrinogen (g/L) | 2.4 [1.91-3.12] | 2.2 [1.67-3.39] | 0.857 | 1.98 [1.38-2.22] | 2.05 [1.73-2.42] | 0.704 |
| Ferritin (ng/L) | 7791 [3686-25928] | 3359 [2654-16500] | 0.174 | 6397 [4015-12107] | 3726 [2834-14308] | 0.428 |
| Triglycerides (mmol/L) | 3.4 [2.42-3.95] | 3.02 [1.7-5.8] | 0.918 | 3.51 [2.66-3.83] | 2.3 [1.75-5.15] | 0.515 |
| Sodium (mEq/L) | 135 [130-139] | 135 [130-136] | 0.643 | 137 [131-140] | 132 [129-134] | 0.210 |
| **HScore** | 243 [236-254] | 205 [154-223] | **0.031** | 252 [245-276] | 198 [172-242] | 0.073 |
| **MAS treatment** |  |  |  |  |  |  |
| Steroid pulses | 12 (92) | 11 (85) | >0.99 | 5 (83) | 8 (80) | >0.99 |
| Anakinra | 9 (69) | 6 (46) | 0.428 | 5 (83) | 3 (30) | 0.119 |
| Tocilizumab | 2 (15) | 0 (0) | 0.480 | 1 (17) | 0 (0) | 0.375 |
| Cyclophosphamide | 0 (0) | 2 (15) | 0.480 | 0 (0) | 1 (10) | >0.99 |
| IVIG | 5 (39) | 5 (39) | >0.99 | 3 (50) | 4 (40) | >0.99 |
| CNIs | 7 (54) | 7 (54) | >0.99 | 4 (67) | 4 (40) | 0.608 |
| Rituximab | 0 (0) | 5 (39) | **0.039** | 0 (0) | 2 (20) | 0.500 |
| Plasma exchange | 4 (31) | 5 (39) | >0.99 | 1 (17) | 2 (20) | >0.99 |
| Etoposide | 0 (0) | 2 (15) | 0.480 | 0 (0) | 0 (0) | - |

*ALP: alkaline phosphatase; ALT: alanine aminotransferase; AST: aspartate aminotransferase; CNIs: calcineurin inhibitors; CNS: central nervous system; CRP: C-reactive protein; ICU: intensive care unit; IVIG: intravenous immunoglobulins; LDH: lactate dehydrogenase; PT: prothrombin time; WBC: white blood cells.*

**Supplementary Table S4.** Univariable analysis exploring putative associations between MAS features, ICU admission and MAS-related death in patients with SLE and SD.

|  | **Associations with ICU admission** | | | | **Associations with MAS-related death** | | | |
| --- | --- | --- | --- | --- | --- | --- | --- | --- |
|  | **SLE** | | **SD** | | **SLE** | | **SD** | |
| **Variable** | **OR (95% CI)** | **p** | **OR (95% CI)** | **p** | **OR (95% CI)** | **p** | **OR (95% CI)** | **p** |
| Male sex | 2.86 (0.71-12.5) | 0.145 | 2.33 (0.59-9.68) | 0.229 | 6.70 (1.45-33.3) | **0.020** | 3.64 (0.61-29.41) | 0.171 |
| Age at MAS | 1.01 (0.97-1.05) | 0.651 | 1.04 (1.01-1.09) | **0.029** | 1.10 (1.04-1.19) | **0.004** | 1.06 (1.01-1.12) | **0.024** |
| Hemorrhage | 2.22 (0.44-11.5) | 0.326 | 10.2 (1.30-215) | **0.050** | 3.83 (0.72-21.2) | 0.111 | 60.0 (5.88-1574) | **0.002** |
| CNS dysfunction | 38.50 (6.72-356) | **<0.001** | 4.89 (0.81-40.1) | 0.096 | 14.0 (2.69-112) | **0.004** | 29.0 (3.65-359) | **0.003** |
| Hepatomegaly | 1.43 (0.36-5.71) | 0.609 | 0.66 (0.16-2.82) | 0.565 | 0.97 (0.21-4.23) | 0.967 | 0.95 (0.16-7.70) | 0.959 |
| Splenomegaly | 1.43 (0.36-5.71) | 0.609 | 0.59 (0.13-2.89) | 0.504 | 1.70 (0.39-7.61) | 0.478 | NA | NA |
| Lymphadenopathy | 0.70 (0.18-2.80) | 0.609 | 0.88 (0.18-4.98) | 0.874 | 1.03 (0.24-4.84) | 0.967 | NA | NA |
| Pneumonitis | 19.71 (2.75-408) | **0.010** | 17.6 (3.28-145) | **0.002** | 12.5 (2.09-107) | **0.009** | 8.33 (1.32-71.6) | **0.030** |
| Hemophagocytosis | 1.69 (0.35-7.98) | 0.504 | 3.20 (0.81-13.9) | 0.104 | 0.71 (0.09-3.78) | 0.710 | 9.09 (1.26-186) | **0.057** |
| Infection | 8.55 (1.98-44.5) | **0.006** | 6.08 (1.44-29.5) | **0.018** | 6.67 (1.44-38.3) | **0.020** | 14.4 (1.94-299) | **0.023** |
| WBC (cells/mm^3^) | 1.00 (1.00-1.00) | 0.977 | 1.00 (1.00-1.00) | 0.364 | 1.00 (1.00-1.00) | 0.168 | 1.00 (1.00-1.00) | 0.176 |
| Hemoglobin (g/dL) | 0.76 (0.47-1.17) | 0.232 | 0.65 (0.39-0.97) | **0.059** | 1.01 (0.64-1.58) | 0.972 | 0.71 (0.36-1.15) | 0.222 |
| Platelets (cells/mm^3^) | 0.98 (0.96-0.99) | **0.012** | 1.00 (0.99-1.00) | 0.984 | 0.98 (0.96-0.99) | **0.019** | 0.99 (0.97-1.00) | 0.095 |
| AST (IU/L) | 1.00 (0.99-1.01) | 0.702 | 1.00 (1.00-1.00) | 0.306 | 1.00 (0.99-1.01) | 0.780 | 1.00 (1.00-1.00) | 0.213 |
| ALT (IU/L) | 1.00 (0.99-1.01) | 0.664 | 1.00 (1.00-1.00) | 0.345 | 1.00 (0.99-1.01) | 0.845 | 1.00 (1.00-1.00) | 0.223 |
| Creatinine (mg/dL) | 6.08 (1.60-36.8) | **0.024** | 22.2 (2.68-432) | **0.015** | 5.12 (1.46-28.2) | **0.031** | 9.33 (1.22-118) | **0.044** |
| CRP (mg/L) | 1.00 (1.00-1.01) | 0.704 | 1.00 (0.99-1.00) | 0.805 | 1.00 (0.99-1.01) | 0.820 | 1.00 (0.99-1.01) | 0.670 |
| Fibrinogen (g/L) | 0.74 (0.38-1.28) | 0.320 | 0.96 (0.64-1.35) | 0.806 | 0.44 (0.15-0.94) | 0.071 | 0.65 (0.27-1.16) | 0.244 |
| Ferritin (ng/L) | 1.00 (1.00-1.00) | 0.082 | 1.00 (1.00-1.00) | 0.533 | 1.00 (1.00-1.00) | 0.222 | 1.00 (1.00-1.00) | 0.935 |
| Triglycerides (mmol/L) | 1.11 (0.90-1.43) | 0.351 | 1.20 (0.84-1.77) | 0.326 | 1.03 (0.79-1.28) | 0.792 | 1.24 (0.80-1.92) | 0.297 |
| Sodium (mEq/L) | 0.95 (0.83-1.08) | 0.409 | 0.92 (0.78-1.05) | 0.295 | 0.83 (0.68-0.97) | **0.030** | 0.99 (0.82-1.13) | 0.860 |
| HScore | 1.00 (0.99-1.02) | 0.565 | 1.01 (0.99-1.03) | 0.216 | 1.01 (0.99-1.02) | 0.368 | 1.03 (1.00-1.06) | 0.081 |

**Supplementary Table S5**. Comparison of MAS characteristics between inaugural and non-inaugural cases in the whole cohort and within the two disease subgroups (SLE and SD).

| **Variable** | **Overall MAS, inaugural** | **Overall MAS, non-inaugural** | **p** | **SLE-MAS, inaugural** | **SLE-MAS, non-inaugural** | **p** | **SD-MAS, inaugural** | **SD-MAS, non-inaugural** | **p** |
| --- | --- | --- | --- | --- | --- | --- | --- | --- | --- |
| Disease | SLE: 18/40 (45) | SLE: 19/34 (56) | 0.484 |  |  |  |  |  |  |
|  | SD: 22/40 (55) | SD: 15/34 (44) |  |  |  |  |  |  |  |
| **Demographic features** |  |  |  |  |  |  |  |  |  |
| Age at disease onset | 26 (15–43) | 26.5 (19–52) | 0.484 | 31 (24–53) | 32 (24–47) | 0.879 | 18 (9–37) | 22 (7–55) | 0.467 |
| Age at MAS | 26 (15–43) | 43 (27–57) | **0.008** | 31 (24–53) | 43 (28–56) | 0.456 | 18 (9–37) | 34 (27–56) | **0.013** |
| Sex | 28/40 (70) | 17/34 (50) | 0.129 | 13/18 (72) | 10/19 (53) | 0.374 | 15/22 (68) | 7/15 (47) | 0.333 |
| Death | 5/40 (12.5) | 14/34 (41) | **0.011** | 4/18 (22) | 9/19 (47) | 0.170 | 1/22 (4.5) | 5/15 (33) | **0.031** |
| Need for ICU | 11/40 (27.5) | 15/34 (44) | 0.212 | 7/18 (39) | 6/19 (32) | 0.904 | 4/22 (18) | 9/15 (60) | **0.015** |
| Death due to MAS | 4/40 (10) | 12/34 (35) | **0.011** | 3/18 (17) | 7/19 (37) | 0.269 | 1/22 (4.5) | 5/15 (33) | **0.031** |
| Follow-up duration | 5 (1–10) | 5 (1–11) | 0.794 | 3 (1–10) | 5 (2–9) | 0.625 | 6 (2–8) | 4 (0.5–14) | 0.975 |
| **MAS phenotype** |  |  |  |  |  |  |  |  |  |
| Fever | 40/40 (100) | 33/34 (97) | 0.460 | 18/18 (100) | 18/19 (94) | >0.99 | 22/22 (100) | 15/15 (100) | >0.99 |
| Hemorrhage | 4/40 (10) | 9/34 (26.5) | 0.075 | 3/18 (17) | 5/19 (26) | 0.693 | 1/22 (4.5) | 4/15 (27) | 0.136 |
| CNS dysfunction | 10/40 (25) | 10/34 (29) | 0.870 | 7/18 (39) | 7/19 (37) | >0.99 | 3/22 (14) | 3/15 (20) | 0.670 |
| Hepatomegaly | 20/40 (50) | 20/34 (59) | 0.600 | 7/18 (39) | 8/19 (42) | >0.99 | 13/22 (59) | 12/15 (80) | 0.286 |
| Splenomegaly | 21/40 (52.5) | 22/34 (65) | 0.410 | 5/18 (28) | 10/19 (53) | 0.229 | 16/22 (73) | 12/15 (80) | 0.711 |
| Lymphadenopathy | 27/40 (67.5) | 24/34 (71) | 0.973 | 11/18 (61) | 11/19 (58) | >0.99 | 16/22 (73) | 13/15 (87) | 0.431 |
| Pneumonitis | 6/40 (15) | 11/34 (32) | 0.136 | 3/18 (17) | 4/19 (21) | >0.99 | 3/22 (14) | 7/15 (47) | **0.056** |
| Myocarditis | 2/40 (5) | 2/34 (5.9) | >0.99 | 1/18 (5.6) | 1/19 (5.3) | >0.99 | 1/22 (4.5) | 1/15 (6.7) | >0.99 |
| Hemophagocytosis | 14/40 (35) | 11/34 (32) | >0.99 | 4/18 (22) | 5/19 (26) | >0.99 | 10/22 (46) | 6/15 (40) | >0.99 |
| Any infection | 9/40 (22.5) | 18/34 (53) | **0.014** | 5/18 (28) | 9/19 (47) | 0.374 | 4/22 (18) | 9/15 (60) | **0.015** |
| **MAS chemistries** |  |  |  |  |  |  |  |  |  |
| WBC (cells/mm^3^) | 5050 (3103–14203) | 3780 (1955–6703) | **0.040** | 3215 (1908–5278) | 3660 (1820–4960) | >0.99 | 10340 (4475–18268) | 4100 (2250–8140) | **0.015** |
| Neutrophils (cells/mm^3^) | 3150 (1588–7425) | 1835 (908–3820) | **0.043** | 2047 (1138–3253) | 1900 (1255–3420) | >0.99 | 5950 (2823–14043) | 1600 (410–4195) | **0.013** |
| Lymphocytes (cells/mm^3^) | 840 (552–1775) | 565 (268–1050) | **0.057** | 585 (325–840) | 540 (275–800) | 0.682 | 1530 (743–4373) | 772 (270–2335) | 0.111 |
| Hemoglobin (g/dL) | 9.1 (7.9–10.0) | 9.1 (7.8–10.1) | 0.970 | 8.7 (7.3–9.5) | 9.1 (7.6–10.0) | 0.186 | 9.7 (8.9–11.1) | 9.5 (8.1–10.9) | 0.653 |
| Platelets (cells/mm^3^) | 107 (80–183) | 97 (40–153) | 0.126 | 94 (68–148) | 108 (54–151) | 0.715 | 121 (92–213) | 48 (13–145) | **0.020** |
| AST (IU/L) | 132 (85–271) | 86 (55–145) | **0.018** | 110 (65–186) | 86 (56–141) | 0.438 | 191 (107–469) | 85 (54–209) | **0.040** |
| ALT (IU/L) | 116 (65–244) | 97 (58–146) | 0.281 | 72 (48–112) | 77 (54–131) | 0.553 | 180 (98–396) | 117 (79–165) | 0.115 |
| ALP (IU/L) | 136 (82–211) | 179 (97–407) | 0.186 | 82 (49–152) | 134 (71–417) | 0.149 | 164.5 (113–217) | 192 (155–378) | 0.337 |
| LDH (IU/L) | 620 (399–905) | 681 (455–817) | 0.833 | 507 (347–800) | 587 (305–765) | 0.761 | 800 (442–1925) | 780 (575–1604) | 0.745 |
| Creatinine (mg/dL) | 0.73 (0.59–0.97) | 0.95 (0.61–1.2) | 0.114 | 0.78 (0.66–1.27) | 1.01 (0.63–1.3) | 0.693 | 0.7 (0.5–0.9) | 0.9 (0.62–1.15) | 0.091 |
| CRP (mg/L) | 57 (30.9–177) | 77 (33–213) | 0.645 | 33 (9–75) | 51 (15.5–110) | 0.616 | 90.9 (49.5–188) | 170 (40–267) | 0.643 |
| Fibrinogen (g/L) | 2.5 (1.6–3.3) | 2.4 (1.9–3.7) | 0.733 | 2.5 (1.8–3.3) | 2.4 (1.9–3.6) | 0.727 | 2.6 (1.4–3.8) | 2.4 (1.5–3.8) | 0.889 |
| Ferritin (ng/L) | 7172 (3132–16444) | 5145 (2753–10597) | 0.368 | 3387 (2282–6891) | 4092 (2302–12115) | 0.620 | 13273 (6675–24506) | 7029 (3293–10446) | 0.142 |
| Triglycerides (mmol/L) | 2.9 (2.2–4.0) | 2.4 (2.0–4.8) | 0.501 | 2.9 (2.1–3.0) | 2.3 (1.8–5.3) | 0.832 | 3.1 (2.4–4.5) | 2.6(2.3–3.8) | 0.536 |
| Sodium (mEq/L) | 135 (131–138) | 136 (131–140) | 0.931 | 136 (130–138) | 135 (131–140) | 0.617 | 135 (131–139) | 138 (134–139) | 0.608 |
| **HScore** | 206 (167–232) | 230 (185–253) | 0.112 | 175 (146–206) | 204 (182–236) | **0.064** | 225 (201–237) | 244 (229–270) | **0.049** |
| **Classified as MAS** | 39/40 (97.5) | 32/34 (94) | 0.591 | 17/18 (94) | 17/19 (90) | >0.99 | 22/22 (100) | 15/15 (100) | >0.99 |
| **MAS treatment** |  |  |  |  |  |  |  |  |  |
| Steroid pulses | 34/40 (85) | 30/34 (88) | 0.745 | 16/18 (89) | 16/19 (84) | >0.99 | 18/22 (82) | 14/15 (93) | 0.629 |
| Anakinra | 18/40 (45) | 9/34 (29) | 0.159 | 6/18 (33) | 1/19 (5.3) | **0.042** | 12/22 (55) | 8/15 (53) | >0.99 |
| Other IL-1 antagonists | 2/40 (5) | 1/34 (2.9) | >0.99 |  |  |  | 2/22 (9.1) | 1/15 (6.7) | >0.99 |
| Tocilizumab | 2/40 (5) | 1/34 (2.9) | >0.99 |  |  |  | 2/22 (9.1) | 1/15 (6.7) | >0.99 |
| Cyclophosphamide | 4/40 (10) | 2/34 (5.9) | 0.681 | 2/18 (11) | 1/19 (5.3) | 0.604 | 2/22 (9.1) | 1/15 (6.7) | >0.99 |
| IVIG | 10/40 (25) | 10/34 (29) | 0.870 | 6/18 (33) | 6/19 (32) | >0.99 | 4/22 (18) | 4/15 (27) | 0.690 |
| CNIs | 16/40 (40) | 22/34 (65) | **0.059** | 7/18 (39) | 10/19 (53) | 0.611 | 9/22 (41) | 12/15 (80) | **0.041** |
| Rituximab | 7/40 (17.5) | 5/34 (15) | 0.993 | 7/18 (39) | 5/19 (26) | 0.642 |  |  |  |
| Plasma exchange | 8/40 (20) | 3/34 (8.8) | 0.208 | 6/18 (33) | 1/19 (5.3) | **0.042** | 2/22 (9.1) | 2/15 (13) | >0.99 |
| TNF inhibitors | 0/40 (0) | 1/34 (2.9) | 0.460 |  |  |  | 0/22 (0) | 1/15 (6.7) | 0.405 |
| Emapalumab | 1/40 (2.5) | 0/34 (0) | >0.99 |  |  |  | 1/22 (4.5) | 0/15 (0) | >0.99 |
| Etoposide | 2/40 (5) | 0/34 (0) | 0.497 | 2/18 (11) | 0/19 (0) | 0.230 | 0/22 (0) | 0/15 (0) | >0.99 |

*ALP: alkaline phosphatase; ALT: alanine aminotransferase; AST: aspartate aminotransferase; CNIs: calcineurin inhibitors; CNS: central nervous system; CRP: C-reactive protein; ICU: intensive care unit; IVIG: intravenous immunoglobulins; LDH: lactate dehydrogenase; PT: prothrombin time; WBC: white blood cells*

**Supplementary Table S6**. Comparison of the underlying disease phenotype in patients with SLE-MAS and SD-MAS according to the timing of MAS (i.e., inaugural vs. non-inaugural).

| **Variable** | **SLE-MAS inaugural** | **SLE-MAS non-inaugural** | **p** | **Variable** | **SD-MAS inaugural** | **SD-MAS non-inaugural** | **p** |
| --- | --- | --- | --- | --- | --- | --- | --- |
| **SLE phenotype** |  |  |  | **SD phenotype** |  |  |  |
| Acute cutaneous lupus | 12/18 (67) | 15/19 (79) | 0.476 | Fever | 21/22 (96) | 15/15 (100) | >0.99 |
| Subacute cutaneous lupus | 5/18 (28) | 8/19 (42) | 0.570 | Arthralgia | 21/22 (96) | 13/15 (87) | 0.554 |
| Discoid lupus | 0/18 (0) | 2/19 (11) | 0.487 | Arthritis | 17/22 (77) | 10/15 (67) | 0.737 |
| Oral ulcers | 8/18 (44) | 6/19 (32) | 0.640 | Myalgia | 14/22 (64) | 8/15 (53) | 0.775 |
| Alopecia | 2/18 (11) | 7/19 (37) | 0.125 | Rash | 21/22 (96) | 8/15 (53) | **0.004** |
| Arthritis | 12/18 (67) | 14/19 (74) | 0.915 | Sore throat | 15/22 (68) | 7/15 (47) | 0.333 |
| Myositis | 4/18 (22) | 4/19 (21) | >0.99 | Abdominal pain | 12/22 (55) | 8/15 (53) | >0.99 |
| Nephritis | 2/18 (11) | 7/19 (37) | 0.125 | Lymphadenopathy | 15/22 (68) | 12/15 (80) | 0.481 |
| Pericarditis | 3/18 (17) | 3/19 (16) | >0.99 | Hepatomegaly | 11/22 (50) | 12/15 (80) | 0.091 |
| Psychosis | 2/18 (11) | 0/19 (0) | 0.230 | Splenomegaly | 17/22 (77) | 11/15 (73) | >0.99 |
| Seizures | 2/18 (11) | 0/19 (0) | 0.230 | Hepatitis | 19/22 (86) | 12/15 (80) | 0.670 |
| Headaches | 4/18 (22) | 6/19 (32) | 0.714 | Pleurisy | 3/22 (14) | 8/15 (53) | **0.025** |
| Raynaud's phenomenon | 2/18 (11) | 7/19 (37) | 0.125 | Pericarditis | 3/22 (14) | 5/15 (33) | 0.228 |
| Sicca syndrome | 2/18 (11) | 5/19 (26) | 0.405 | Inflammatory anemia | 17/22 (77) | 13/15 (87) | 0.677 |
| Anemia | 16/18 (89) | 13/19 (68) | 0.232 | WBC > 10,000/mmc | 13/22 (59) | 13/15 (87) | 0.141 |
| Direct Coombs test | 13/14 (93) | 6/12 (50) | **0.026** | WBC > 15,000/mmc | 14/22 (64) | 11/15 (73) | 0.724 |
| Leukopenia | 7/18 (39) | 10/19 (53) | 0.611 | Neutrophilia | 14/22 (64) | 14/15 (93) | **0.056** |
| Lymphopenia | 14/18 (78) | 13/19 (68) | 0.714 | High CRP | 21/22 (96) | 15/15 (100) | >0.99 |
| Thrombocytopenia | 12/18 (67) | 10/19 (53) | 0.593 | Hyperferritinemia | 21/22 (96) | 14/15 (93) | >0.99 |
| Low complement C3 | 11/18 (61) | 11/19 (58) | >0.99 | Pneumonitis | 1/22 (4.5) | 5/15 (33) | **0.031** |
| Low complement C4 | 9/18 (50) | 12/19 (63) | 0.634 | Lung fibrosis | 0/22 (0) | 3/15 (20) | **0.059** |
| **Serologies** |  |  |  |  |  |  |  |
| ANA | 18/18 (100) | 19/19 (100) | 0.869 |  |  |  |  |
| anti-dsDNA | 11/18 (61) | 13/19 (68) | 0.904 |  |  |  |  |
| anti-SSA | 10/18 (56) | 8/19 (42) | 0.625 |  |  |  |  |
| anti-SSB | 2/18 (11) | 2/19 (11) | >0.99 |  |  |  |  |
| anti-U1RNP | 5/18 (28) | 10/19 (53) | 0.229 |  |  |  |  |
| anti-Sm | 5/18 (28) | 8/19 (42) | 0.570 |  |  |  |  |
| anti-Ribosomal P | 2/18 (11) | 2/19 (11) | >0.99 |  |  |  |  |
| Lupus anticoagulant | 4/18 (22) | 5/19 (26) | >0.99 |  |  |  |  |
| **SLE treatment** |  |  |  | **SD treatment** |  |  |  |
| Glucocorticoids | 18/18 (100) | 17/19 (90) | 0.487 | Glucocorticoids | 22/22 (100) | 15/15 (100) | >0.99 |
| Methotrexate | 3/18 (17) | 4/19 (21) | >0.99 | Methotrexate | 6/22 (27) | 7/15 (47) | 0.388 |
| CNIs | 4/18 (22) | 7/19 (37) | 0.476 | CNIs | 7/22 (32) | 7/15 (47) | 0.569 |
| Cyclophosphamide | 3/18 (17) | 1/19 (5.3) | 0.340 | Anakinra | 14/22 (64) | 6/15 (40) | 0.280 |
| Azathioprine | 2/18 (11) | 4/19 (21) | 0.660 | Other IL-1 antagonists | 6/22 (27) | 2/15 (13) | 0.431 |
| Mycophenolate mofetil | 10/18 (56) | 6/19 (32) | 0.255 | Tocilizumab | 8/22 (36) | 4/15 (27) | 0.724 |
| Rituximab | 8/18 (44) | 7/19 (37) | 0.892 | JAK inhibitors | 2/22 (9.1) | 1/15 (6.7) | >0.99 |
| Belimumab | 3/18 (17) | 3/19 (16) | >0.99 |  |  |  |  |
| Anifrolumab | 0/18 (0) | 1/19 (5.3) | >0.99 |  |  |  |  |
| Hydroxychloroquine | 14/18 (78) | 13/19 (68) | 0.714 |  |  |  |  |

**Supplementary Table S7**. Stability of LDA coefficients assessed by non-parametric bootstrap resampling (1000 resamples with replacement). For each variable included in the multivariate model, the mean LD1 weight, 95% bootstrap confidence interval, and sign consistency (proportion of bootstrap resamples in which the coefficient sign matched that of the full-dataset model) are reported, together with the direction of association (SLE-MAS or SD-MAS).

| **Variable** | **Direction** | **LD1 (95%CI)** | **Sign consistency** |
| --- | --- | --- | --- |
| ALT | Still's | -2.45 (-6.53 to 1.19) | 91.6% |
| AST | SLE | 2.03 (-1.72 to 6.14) | 86.7% |
| Myocardial damage | SLE | 1.99 (-2.34 to 6.15) | 85.7% |
| Ferritin | Still's | -1.33 (-2.53 to -0.35) | 99.3% |
| Pneumonitis | Still's | -1.29 (-4.08 to 1.09) | 86.9% |
| Sex | SLE | 1.19 (-0.30 to 3.02) | 95.1% |
| Splenomegaly | Still's | -1.00 (-2.59 to 0.49) | 91.5% |
| Lymphadenopathy | SLE | 0.87 (-1.03 to 2.86) | 82.4% |
| Hepatomegaly | Still's | -0.87 (-2.56 to 0.73) | 86.2% |
| Lymphocyte count | Still's | -0.86 (-1.70 to -0.09) | 98.5% |
| Sodium | Still's | -0.69 (-1.91 to 0.27) | 93.0% |
| CRP | Still's | -0.62 (-1.56 to 0.05) | 96.3% |
| Infection | SLE | 0.53 (-1.76 to 2.83) | 73.2% |
| ALP | SLE | 0.48 (-0.75 to 1.41) | 82.3% |
| Hb | Still's | -0.44 (-1.31 to 0.36) | 88.0% |
| Hemorrhage | Still's | -0.43 (-3.15 to 1.75) | 62.4% |
| Creatinine | SLE | 0.38 (-0.76 to 1.21) | 83.7% |
| CNS involvement | SLE | 0.35 (-1.53 to 2.12) | 64.8% |
| Neutrophil count | Still's | -0.31 (-1.38 to 0.63) | 73.6% |
| Age at MAS | SLE | 0.22 (-0.74 to 1.24) | 68.2% |
| PT ratio | SLE | 0.20 (-4.42 to 3.79) | 69.9% |
| Bone marrow hemophagocytosis | SLE | 0.18 (-1.62 to 2.05) | 57.5% |
| Fever | Still's | -0.14 (-2.82 to 2.63) | 55.1% |
| Inaugural MAS | Still's | -0.14 (-2.03 to 1.54) | 54.7% |
| PLT count | SLE | 0.11 (-0.69 to 1.05) | 56.3% |
| Fibrinogen | SLE | 0.10 (-0.69 to 1.14) | 57.3% |
| LDH | Still's | -0.07 (-1.88 to 1.70) | 53.8% |
| Triglycerides | Still's | -0.03 (-1.10 to 0.84) | 47.6% |

**Supplementary Table S8**. Results of the systematic literature case retrieval of previous reports of SLE-MAS included in our analysis.

Barlas N, Kaur IP, Kuzhuppilly Arcilla C, Kaeley GS, Thway M. Effective Treatment of Macrophage Activation Syndrome Linked to Systemic Lupus Erythematosus Using Interleukin-1 Inhibitor. Cureus. 2025 Apr 16;17(4):e82379. doi: 10.7759/cureus.82379. PMID: 40385895; PMCID: PMC12083551.

Alharbi LK, Ahmed AK. Haemophagocytic Lymphohistiocytosis as an Initial Presentation of Undiagnosed Systemic Lupus Erythematosus. Eur J Case Rep Intern Med. 2025 Apr 14;12(5):005336. doi: 10.12890/2025_005336. PMID: 40352701; PMCID: PMC12061209.

Rakotonirina LN, Andrianiaina MO, Rakotonirainy OH, Ramanandafy H, Randriamifidy NH, Rapelanoro Rabenja F. Management Challenge of Coexistence of Macrophage Activation Syndrome, Systemic Lupus Erythematosus, and Hepatitis B: Case Report. Clin Case Rep. 2025 Apr 10;13(4):e70419. doi: 10.1002/ccr3.70419. PMID: 40226232; PMCID: PMC11985889.

Chiara E, Gasparotto M, Malandrino D, Biancalana E, Uban ML, Silvestri E, Emmi G. Systemic lupus erythematosus-related macrophage activation syndrome inducing severe cytopenia successfully treated with anifrolumab: a case report. Clin Exp Rheumatol. 2025 Jan 24. doi: 10.55563/clinexprheumatol/uumehz. Epub ahead of print. PMID: 39907615.

Gao SP, Luo XF, Kosari M, Li WJ, Yang L, Tu W, Zhong JX. Successful management of infection and macrophage activation syndrome patient using low-dose etoposide: A case report. World J Radiol. 2024 Oct 28;16(10):579-585. doi: 10.4329/wjr.v16.i10.579. PMID: 39494131; PMCID: PMC11525825.

Gohri J, Satish S, Muniz JP, Arellano M. EBV-triggered secondary macrophage activation syndrome in the backdrop of lupus in an adult. Indian J Pathol Microbiol. 2024 Sep 18. doi: 10.4103/ijpm.ijpm_221_24. Epub ahead of print. PMID: 39291492.

Ahmad A, Atluri R, Robbins KJ. Hemophagocytic Lymphohistiocytosis Unmasking Systemic Lupus Erythematosus: Management With Belimumab and a Case Study. Cureus. 2024 Jul 15;16(7):e64596. doi: 10.7759/cureus.64596. PMID: 39149687; PMCID: PMC11325255.

Shaykh N, Rai O, Karan A, Bhullar KK, Tripathi V, Reddy P. Unleashing the Cytokine Storm: A Case of Macrophage Activation Syndrome in Systemic Lupus Erythematosus. Cureus. 2024 Jun 25;16(6):e63167. doi: 10.7759/cureus.63167. PMID: 39070482; PMCID: PMC11272981.

Sara M, Anna S, Lorenzo B, Valentina B, Giuseppe C. Macrophage activation syndrome secondary to systemic lupus erythematosus: lesson for the clinical nephrologist. J Nephrol. 2024 Nov;37(8):2379-2382. doi: 10.1007/s40620-024-01997-6. Epub 2024 Jun 28. PMID: 38940999.

Abdelfattah AH, Macpherson A, Javed F. Macrophage Activation Syndrome Presents as Initial Manifestation of Lupus in an Adult Female. Cureus. 2024 May 18;16(5):e60567. doi: 10.7759/cureus.60567. PMID: 38894758; PMCID: PMC11184542.

Kuna J, Chmielewski G, Jaśkiewicz Ł, Krajewska-Włodarczyk M. Acute Heart Failure in the Course of Macrophage Activation Syndrome Due to Newly Diagnosed Systemic Lupus Erythematosus: Case Presentation and Literature Review. Medicina (Kaunas). 2024 Feb 26;60(3):392. doi: 10.3390/medicina60030392. PMID: 38541118; PMCID: PMC10971827.

Ramchandran R, Cuesta B, Kathari YK, Kallen ME, McCool I, Singh ZN, Law JY, Baer MR. Macrophage activation syndrome after granulocyte colony-stimulating factor in a patient with systemic lupus erythematosus. Br J Haematol. 2024 Mar;204(3):747-748. doi: 10.1111/bjh.19262. Epub 2023 Dec 22. PMID: 38135898.

Arya P V A, Alam MM, Bernhisel A, Degirolamo A, Huang R. Diagnostic Dilemma in a Case of Necrotizing Lymphadenitis With Macrophage Activation Syndrome. Cureus. 2023 Jul 21;15(7):e42267. doi: 10.7759/cureus.42267. PMID: 37605695; PMCID: PMC10440125.

Fioravante da Silveira L, Zago A, Zanchet D, Chemello D. Macrophage activation syndrome in a patient with systemic lupus erythematosus undergoing cyclophosphamide treatment: a case report. Reumatismo. 2023 Jul 17;75(2). doi: 10.4081/reumatismo.2023.1597. PMID: 37462126.

Abughazaleh S, Alsakarneh S, Hattar M, Jaber F, Palmer Q, Ghoz H. Systemic Lupus Erythematosus in Disguise: Acute Pancreatitis as Initial Presentation. J Investig Med High Impact Case Rep. 2023 Jan-Dec;11:23247096231185480. doi: 10.1177/23247096231185480. PMID: 37421299; PMCID: PMC10331329.

Phoophiboon V, Brown P, Burns KEA. Systemic lupus erythematosus associated with development of macrophage activation syndrome and disseminated aspergillosis. Can J Anaesth. 2023 Jul;70(7):1255-1260. English. doi: 10.1007/s12630-023-02506-2. Epub 2023 Jun 22. PMID: 37349668.

Sengupta D, Poddar K, Bag U, Ghosh S. Reversible blindness with macrophage activation syndrome: an unusual presentation of secondary antiphospholipid syndrome. BMJ Case Rep. 2023 Apr 4;16(4):e253317. doi: 10.1136/bcr-2022-253317. PMID: 37015764; PMCID: PMC10083791.

Sukhadiya P, Kumar P, Meena DS, Kumar P H A, Vijayan N, Garg P, Garg MK. Unmasking of systemic lupus erythematosus in a patient with hemophagocytic lymphohistiocytosis- macrophage activation syndrome (HLA-MAS) and diffuse alveolar hemorrhage. Reumatismo. 2023 Mar 21;74(4). doi: 10.4081/reumatismo.2022.1488. PMID: 36942984.

Ikić Matijašević M, Kilić P, Ikić L, Galić I, Brzović Šarić V, Galić E. Rituximab, Intravitreal Bevacizumab and Laser Photocoagulation for Treatment of Macrophage Activation Syndrome and Retinal Vasculitis in Lupus: A Case Report. Int J Mol Sci. 2023 Jan 30;24(3):2594. doi: 10.3390/ijms24032594. PMID: 36768956; PMCID: PMC9916420.

Yamaguchi M, Mizuno M, Kitamura F, Iwagaitsu S, Nobata H, Kinashi H, Banno S, Asai A, Ishimoto T, Katsuno T, Ito Y. Case report: Thrombotic microangiopathy concomitant with macrophage activation syndrome in systemic lupus erythematosus refractory to conventional treatment successfully treated with eculizumab. Front Med (Lausanne). 2023 Jan 9;9:1097528. doi: 10.3389/fmed.2022.1097528. PMID: 36698804; PMCID: PMC9868404.

Tran DH, Jaggon KS, Mikdashi J, Chow RD, Verceles AC, Sood A. Lamotrigine-Induced Lupus With Aseptic Meningitis and Hemophagocytic Lymphohistiocytosis. Cureus. 2022 Sep 26;14(9):e29629. doi: 10.7759/cureus.29629. PMID: 36176480; PMCID: PMC9512686.

Suwal A, Shrestha B, Paudel A, Paudel R, Basnet S. A Case of Macrophage Activation Syndrome Manifesting as the Initial Presentation of Systemic Lupus Erythematosus. Cureus. 2022 Jun 27;14(6):e26375. doi: 10.7759/cureus.26375. PMID: 35911271; PMCID: PMC9328934.

Kuna J, Chmielewski G, Gruchała M, Szade J, Mikiewicz M, Ręcki P, Krajewska-Włodarczyk M. Fatal Acute Heart Failure in the Course of Macrophage Activation Syndrome: Case Report and Literature Review. J Clin Med. 2022 Jul 20;11(14):4208. doi: 10.3390/jcm11144208. PMID: 35887970; PMCID: PMC9324855.

Vogel C, Manwani P, Cornford ME, Heinze ER. Nemaline myopathy in newly diagnosed systemic lupus erythematosus and Sjögren's overlap syndrome complicated by macrophage activation syndrome. BMC Rheumatol. 2022 Mar 15;6(1):21. doi: 10.1186/s41927-022-00246-2. PMID: 35287748; PMCID: PMC8922735.

Chang ES, Yu HH, Wu CE, Chan TM. Acute respiratory distress syndrome associated with macrophage activation syndrome in systemic lupus erythematosus: A case report and literature review. Medicine (Baltimore). 2022 Feb 4;101(5):e28612. doi: 10.1097/MD.0000000000028612. PMID: 35119005; PMCID: PMC8812624.

Gouda W, Alsaqabi F, Moshrif A, Abbas AS, Abdel-Aziz TM, Islam MA. Macrophage activation syndrome triggered by systemic lupus erythematosus flare: successful treatment with a combination of dexamethasone sodium phosphate, intravenous immunoglobulin, and cyclosporine: a case report. J Med Case Rep. 2021 Oct 7;15(1):497. doi: 10.1186/s13256-021-03072-1. PMID: 34620236; PMCID: PMC8495443.

Awasthi S, Upreti S. Macrophage activation syndrome in a patient with systemic lupus erythematosus (SLE) and dual viremia. J Community Hosp Intern Med Perspect. 2020 Sep 3;10(5):470-474. doi: 10.1080/20009666.2020.1787811. PMID: 33235686; PMCID: PMC7671734.

Kübler L, Bittmann I, Kuipers JG. Macrophage activation syndrome triggered by active systemic lupus erythematosus : Successful treatment by interleukin-1 inhibition (anakinra). Z Rheumatol. 2020 Dec;79(10):1040-1045. English. doi: 10.1007/s00393-020-00836-w. PMID: 32804304.

Shah K, Porter A, Takhar G, Reddy V. Case of using mycophenolate in combination with steroids for concurrent macrophage activation syndrome and lupus flare. BMJ Case Rep. 2020 Apr 7;13(4):e231554. doi: 10.1136/bcr-2019-231554. PMID: 32269047; PMCID: PMC7167458.

Tan CL, Yahaya MH, Ahmad NS, Lim CH. Macrophage activation syndrome as an initial presentation of systemic lupus erythematosus with sensorineural hearing loss in a young male patient. BMJ Case Rep. 2020 Mar 12;13(3):e233330. doi: 10.1136/bcr-2019-233330. PMID: 32169986; PMCID: PMC7069256.

Cowley S, Ramakrishnan S. Macrophage activation syndrome in a patient with systemic lupus erythematosus. Pol Arch Intern Med. 2019 Aug 29;129(7-8):535-538. doi: 10.20452/pamw.14953. Epub 2019 Aug 29. PMID: 31469123.

Poudel P, Swe T, Rayancha S. A Rare Case of Macrophage Activation Syndrome Presenting as the First Manifestation of Systemic Lupus Erythematosus. J Investig Med High Impact Case Rep. 2018 Nov 15;6:2324709618812196. doi: 10.1177/2324709618812196. PMID: 30480003; PMCID: PMC6243398.

Luo Y, Wen Y, Arevalo Molina AB, Dahal P, Leuprecht L, Bsrat M. Macrophage Activation Syndrome, Glomerulonephritis, Pericarditis, and Retinal Vasculitis as Initial Presentation of Systemic Lupus Erythematosus. Case Rep Med. 2018 Sep 26;2018:5979386. doi: 10.1155/2018/5979386. PMID: 30356389; PMCID: PMC6178166.

Szulawski R, Kourlas PJ, Antonchak M. Macrophage Activation Syndrome (MAS) in a Recently Released Prisoner with Systemic Lupus Erythematosus (SLE). Am J Case Rep. 2018 Jun 22;19:734-738. doi: 10.12659/AJCR.906154. PMID: 29930239; PMCID: PMC6047585.

Cruz-Pérez F, Vilá S, Ríos G, Vilá LM. Efficacy of Cyclosporine in the Induction and Maintenance of Remission in a Systemic Lupus Erythematosus Patient Presenting with Macrophage-Activating Syndrome. Case Rep Rheumatol. 2018 Jan 15;2018:1961585. doi: 10.1155/2018/1961585. PMID: 29552369; PMCID: PMC5821990.

Aoyama-Maeda N, Horino T, Ichii O, Terada Y. Macrophage activation syndrome associated with systemic lupus erythematosus treated successfully with the combination of steroid pulse, immunoglobulin and tacrolimus. Rom J Intern Med. 2018 Jun 1;56(2):117-121. doi: 10.1515/rjim-2017-0043. PMID: 29099692.

Tulbă D, Balea M, Băicuş C. Macrophage activation syndrome at the onset of glucocorticoid-resistant systemic lupus erythematosus: a case report. Rom J Intern Med. 2018 Mar 1;56(1):67-70. doi: 10.1515/rjim-2017-0035. PMID: 28918421.

Junga Z, Stitt R, Tracy C, Keith M. Novel use of rituximab in macrophage activation syndrome secondary to systemic lupus erythematosus. BMJ Case Rep. 2017 Aug 21;2017:bcr2017221347. doi: 10.1136/bcr-2017-221347. PMID: 28827301; PMCID: PMC5624074.

Granata G, Didona D, Stifano G, Feola A, Granata M. Macrophage Activation Syndrome as Onset of Systemic Lupus Erythematosus: A Case Report and a Review of the Literature. Case Rep Med. 2015;2015:294041. doi: 10.1155/2015/294041. Epub 2015 May 7. PMID: 26064125; PMCID: PMC4439481.

Ma Y, Reddy V, Reilly S. A fatal case of systemic lupus erythematosus complicated by macrophage activation syndrome, portal vein thrombosis and acute colitis. Int J Rheum Dis. 2017 Nov;20(11):1805-1807. doi: 10.1111/1756-185X.12511. Epub 2014 Dec 4. PMID: 25474387.

Tochihara M, Kasai M, Katsumata Y, Sato E, Ishiguro N, Kazama H, Sugimoto N, Ichida H, Kawaguchi Y, Yamanaka H. Erythematosus plaques with macrophage infiltration as an initial manifestation of macrophage activation syndrome in a patient with systemic lupus erythematosus. Mod Rheumatol. 2016 Nov;26(6):956-957. doi: 10.3109/14397595.2014.932042. Epub 2014 Jul 18. PMID: 25036234.

Tayer-Shifman OE, Ben-Chetrit E. Refractory macrophage activation syndrome in a patient with SLE and APLA syndrome - Successful use of PET- CT and Anakinra in its diagnosis and treatment. Mod Rheumatol. 2015;25(6):954-7. doi: 10.3109/14397595.2013.844403. Epub 2013 Oct 21. PMID: 24252009.

Bakshi J, Hassan S, D'Cruz D, Chan A. Rituximab therapy in refractory macrophage activation syndrome secondary to systemic lupus erythematosus. Lupus. 2013 Dec;22(14):1544-6. doi: 10.1177/0961203313504634. Epub 2013 Sep 6. PMID: 24014570.

Kim JM, Kwok SK, Ju JH, Park KS, Park GS, Kim HY, Park SH. Macrophage activation syndrome resistant to medical therapy in a patient with systemic lupus erythematosus and its remission with splenectomy. Rheumatol Int. 2013 Mar;33(3):767-71. doi: 10.1007/s00296-010-1654-4. Epub 2010 Dec 1. PMID: 21120496.

Shah C, Cotter L, Yu E, Wanat K, Harrington A, Sabchyshyn V, Umpierrez A. A Triple Threat: Acute Systemic Lupus Erythematosus Unveiled with Hemophagocytic Lymphohistiocytosis and Toxic Epidermal Necrolysis. J Brown Hosp Med. 2025 Apr 1;4(2):47-53. doi: 10.56305/001c.130831. PMID: 40191701; PMCID: PMC11966765.

Cazzaniga J, Georgiyeva K, Siguenza A, Gonzalez T, Ferraro P. Using Diagnostic Radiological Imaging Modalities to Explore Neurological Dysfunction and Renal Failure in the Intersection of Hemophagocytic Lymphohistiocytosis, Macrophage Activation Syndrome, and Lupus. Cureus. 2024 Feb 11;16(2):e54005. doi: 10.7759/cureus.54005. PMID: 38496127; PMCID: PMC10944546.

Chen Q, Zhang J, Huang H, Qiu T, Jin Z, Shi Y, Zhu H, Fan L, Li J, Shi W, Miao Y. Histiocytic necrotizing lymphadenitis with hemophagocytic lymphohistiocytosis in adults: A single-center analysis of 5 cases. Immun Inflamm Dis. 2024 Feb;12(2):e1202. doi: 10.1002/iid3.1202. PMID: 38411294; PMCID: PMC10898206.

Shao D, Pena O, Sekulic M, Valdez Imbert R, Vegivinti CTR, Jim B. Secondary haemophagocytic lymphohistiocytosis in a patient with new-onset systemic lupus erythematosus: the challenges of timely diagnosis and successful treatment. BMJ Case Rep. 2023 Jul 10;16(7):e252938. doi: 10.1136/bcr-2022-252938. PMID: 37429644; PMCID: PMC10335589.

Fernandes M Sr, Ferreira P, Lynce A, Correia MJ, Ribeiro AM. Post-partum Fever of Unknown Origin: An Inaugural Flare of Severe Lupus With Multisystemic Involvement and Hemophagocytic Syndrome. Cureus. 2023 Jan 4;15(1):e33348. doi: 10.7759/cureus.33348. PMID: 36751216; PMCID: PMC9896851.

Ren W, Yang S, Liu H, Pan Z, Li Z, Qiao P, Ma H. Case report and literature review: Hemophagocytic lymphohistiocytosis in a pregnant woman with systemic lupus erythematosus with *Syntaxin 11* gene defect. Front Oncol. 2022 Jul 28;12:937494. doi: 10.3389/fonc.2022.937494. PMID: 35965579; PMCID: PMC9367683.

Ali R, Mehannek R, Patel A, Paige A, Reddy S, Guma M, Guron G. Systemic Lupus Erythematosus With Hemophagocytic Lymphohistiocytosis: Is COVID-19 the Inciting Factor? Cureus. 2021 Nov 17;13(11):e19657. doi: 10.7759/cureus.19657. PMID: 34976453; PMCID: PMC8679171.

Hardage J, Otto NB, Skaggs J, Travis S. Prompt Recognition of Hemophagocytic Lymphohistiocytosis in an Afebrile Patient with Lupus and Staphylococcus aureus Bacteremia. Am J Case Rep. 2021 Nov 9;22:e934092. doi: 10.12659/AJCR.934092. PMID: 34750340; PMCID: PMC8591524.

Taira M, Matsumura T, Sumita Y, Moriyama M, Kondo M, Ishikawa N, Wada Y, Nagase M, Nishikawa E, Tsubata Y, Kishimoto K, Murakawa Y. A Rare Case of Acute Fibrinous and Organizing Pneumonia Associated with Systemic Lupus Erythematosus and Autoimmune-associated Hemophagocytic Syndrome: The Involvement of CD163-positive Macrophages. Intern Med. 2022 Feb 15;61(4):559-565. doi: 10.2169/internalmedicine.7184-21. Epub 2021 Aug 24. PMID: 34433711; PMCID: PMC8907772.

Li NL, Kang R, Ardoin SP, Ayoub I. Hemophagocytic Lymphohistiocytosis, a Rare Presentation in Lupus Nephritis. Kidney Int Rep. 2020 Dec 13;6(3):842-845. doi: 10.1016/j.ekir.2020.11.036. PMID: 33733000; PMCID: PMC7938066.

Castillo JM, MÁrquez AMB, Cabada IAB. Systemic Lupus Erythematosus and Its Association with Hemophagocytic Syndrome as an Initial Manifestation. Maedica (Bucur). 2020 Dec;15(4):556-560. doi: 10.26574/maedica.2020.15.4.556. PMID: 33603918; PMCID: PMC7879355.

Pradhan S, Sirka CS, Dash G, Sahu K, Rout AN. A challenging case of Kikuchi-Fujimoto disease with systemic lupus erythematosus complicated with hemophagocytic lymphohistiocytosis in a young man. Indian J Dermatol Venereol Leprol. 2021 Jan-Feb;87(1):78-82. doi: 10.25259/IJDVL_893_18. PMID: 33580926.

Tomofuji Y, Ishikawa Y, Hattori K, Fujiwara M, Kita Y. Successful treatment of refractory acute lupus haemophagocytic syndrome using rituximab: a case report. Mod Rheumatol Case Rep. 2020 Jul;4(2):222-228. doi: 10.1080/24725625.2019.1705529. Epub 2020 Jan 6. PMID: 33087000.

Harano Y, Ishikawa Y, Hattori K, Ichinose M, Tomofuji Y, Okano H, Owada G, Kimura Y, Nanao T, Fujimoto J, Nishizawa H, Iioka Y, Osada J, Fujiwara M, Kita Y. A case of complete atrioventricular block in secondary hemophagocytic syndrome/hemophagocytic lymphohistiocytosis recovered by plasma exchange and cytokine absorbing therapy with AN69ST continuous hemodiafiltration. Immunol Med. 2020 Dec;43(4):171-178. doi: 10.1080/25785826.2020.1761145. Epub 2020 May 6. PMID: 32374660.

Ali AH, Mohameden M, Al-Adhami S, Yuvienco C. Cerebral Spinal Fluid Hemophagocytosis in Lupus-Induced Hemophagocytic Lymphohistiocytosis: A Clinical Image. J Clin Rheumatol. 2021 Apr 1;27(3):e84-e85. doi: 10.1097/RHU.0000000000001260. PMID: 31972734.

AlSheef M, Zaidi ARZ, AlAtmi AA, AlSharif LH, Mian A. Hemophagocytic Syndrome as an Initial Presentation of Systemic Lupus Erythematosus: A Case Report. Cureus. 2019 Jul 29;11(7):e5261. doi: 10.7759/cureus.5261. PMID: 31576254; PMCID: PMC6764614.

Thomas M, Robert A, Kuruvilla N, C U. An Unusual Presentation of Systemic Lupus Erythematosus as Hemophagocytic Lymphohistiocytosis in a Male. Cureus. 2019 Aug 19;11(8):e5427. doi: 10.7759/cureus.5427. PMID: 31482047; PMCID: PMC6701905.

Thornton CS, Minoo P, Schneider M, Fifi-Mah A. Severe skin disease in lupus associated with hemophagocytic lymphohistiocytosis: case reports and review of the literature. BMC Rheumatol. 2019 Feb 8;3:7. doi: 10.1186/s41927-019-0055-x. PMID: 30886995; PMCID: PMC6390523.

Gauiran DTV, Cheng PVCY, Pagaduan CRP, Santos MCM. Autoimmune-Associated Hemophagocytosis and Myelofibrosis in a Newly Diagnosed Lupus Patient: Case Report and Literature Review. Case Rep Hematol. 2019 Jan 9;2019:3879148. doi: 10.1155/2019/3879148. PMID: 30729051; PMCID: PMC6343163.

Chokshi B, D'Agati V, Bizzocchi L, Johnson B, Mendez B, Jim B. Haemophagocytic lymphohistiocytosis with collapsing lupus podocytopathy as an unusual manifestation of systemic lupus erythematosus with APOL1 double-risk alleles. BMJ Case Rep. 2019 Jan 14;12(1):bcr-2018-227860. doi: 10.1136/bcr-2018-227860. PMID: 30642866; PMCID: PMC6340519.

Costa A, Pais C, Cerqueira S, Salvador F. Visceral Leishmaniasis Associated with Macrophage Activation Syndrome and Diffuse Alveolar Hemorrhage in a Lupus Patient. Acta Med Port. 2018 Oct 31;31(10):593-596. doi: 10.20344/amp.9028. Epub 2018 Oct 31. PMID: 30387429.

Toko H, Tsuboi H, Umeda N, Honda F, Ohyama A, Takahashi H, Abe S, Yokosawa M, Asashima H, Hagiwara S, Hirota T, Kondo Y, Matsumoto I, Sumida T. Intractable Hemophagocytic Syndrome Associated with Systemic Lupus Erythematosus Resistant to Corticosteroids and Intravenous Cyclophosphamide That Was Successfully Treated with Cyclosporine A. Intern Med. 2018 Sep 15;57(18):2747-2752. doi: 10.2169/internalmedicine.0571-17. Epub 2018 May 18. PMID: 29780126; PMCID: PMC6191589.

Shi W, Duan M, Jie L, Sun W. A successful treatment of severe systemic lupus erythematosus caused by occult pulmonary infection-associated with hemophagocytic syndrome: A case report. Medicine (Baltimore). 2018 May;97(19):e0595. doi: 10.1097/MD.0000000000010595. PMID: 29742693; PMCID: PMC5959410.

Nawata T, Kubo M, Shiragami K, Nakamura Y, Yano M. Successful Treatment of Hemophagocytic Lymphohistiocytosis Associated with Lupus Nephritis by Using Mycophenolate Mofetil. Case Rep Rheumatol. 2017;2017:4159727. doi: 10.1155/2017/4159727. Epub 2017 Sep 14. PMID: 29062579; PMCID: PMC5618750.

Souto Filho JTD, Lima PDA, Paulo AB, Souza ALS. Hemophagocytic lymphohistiocytosis secondary to disseminated histoplasmosis in systemic lupus erythematosus. Int J Hematol. 2017 Dec;106(6):727-728. doi: 10.1007/s12185-017-2308-z. Epub 2017 Aug 7. PMID: 28786081.

Wijetunga WMUA, Satarasinghe RL, Dayananda BM, Darshani GK. Unusual acute lupus hemophagocytic syndrome - a test of diagnostic criteria: a case report. J Med Case Rep. 2017 Jul 7;11(1):185. doi: 10.1186/s13256-017-1339-7. PMID: 28683778; PMCID: PMC5501064.

Gupta D, Mohanty S, Thakral D, Bagga A, Wig N, Mitra DK. Unusual Association of Hemophagocytic Lymphohistiocytosis in Systemic Lupus Erythematosus: Cases Reported at Tertiary Care Center. Am J Case Rep. 2016 Oct 13;17:739-744. doi: 10.12659/ajcr.899433. PMID: 27733745; PMCID: PMC5065291.

Chawki S, Aouizerate J, Trad S, Prinseau J, Hanslik T. Bilateral sudden sensorineural hearing loss as a presenting feature of systemic lupus erythematosus: Case report and brief review of other published cases. Medicine (Baltimore). 2016 Sep;95(36):e4345. doi: 10.1097/MD.0000000000004345. PMID: 27603334; PMCID: PMC5023856.

Sharmeen S, Hussain N. An Unusual Case of Systemic Lupus Erythematosus and Hemophagocytic Syndrome. Case Rep Rheumatol. 2016;2016:8957690. doi: 10.1155/2016/8957690. Epub 2016 Feb 14. PMID: 26981305; PMCID: PMC4769753.

So MW, Koo BS, Kim YJ, Kim YG, Lee CK, Yoo B. Successful rituximab treatment of refractory hemophagocytic lymphohistiocytosis and autoimmune hemolytic anemia associated with systemic lupus erythematosus. Mod Rheumatol. 2014 Sep;24(5):855-7. doi: 10.3109/14397595.2013.874740. Epub 2014 Feb 11. PMID: 24517558.

Ueda Y, Yamashita H, Takahashi Y, Kaneko H, Kano T, Mimori A. Refractory hemophagocytic syndrome in systemic lupus erythematosus successfully treated with intermittent intravenous cyclophosphamide: three case reports and literature review. Clin Rheumatol. 2014 Feb;33(2):281-6. doi: 10.1007/s10067-013-2451-8. Epub 2013 Dec 18. PMID: 24343456.

Jung SY. Hemophagocytic syndrome diagnosed by liver biopsy in a female patient with systemic lupus erythematosus. J Clin Rheumatol. 2013 Dec;19(8):449-51. doi: 10.1097/RHU.0000000000000040. PMID: 24263149.

Hasegawa H, Mizoguchi F, Kohsaka H, Miyasaka N. Systemic lupus erythematosus with cytophagic histiocytic panniculitis successfully treated with high-dose glucocorticoids and cyclosporine A. Lupus. 2013 Mar;22(3):316-9. doi: 10.1177/0961203313476355. PMID: 23439471.

Kikuchi H, Yamamoto T, Asako K, Takayama M, Shirasaki R, Ono Y. Etanercept for the treatment of intractable hemophagocytic syndrome with systemic lupus erythematosus. Mod Rheumatol. 2012 Apr;22(2):308-11. doi: 10.1007/s10165-011-0500-1. Epub 2011 Jul 20. PMID: 21773824.

Watanabe H, Hirase N, Goda H, Nishikawa H, Ikuyama S. Oral low-dose tacrolimus therapy for refractory hemophagocytic syndrome associated with systemic lupus erythematosus. Mod Rheumatol. 2012 Apr;22(2):284-9. doi: 10.1007/s10165-011-0491-y. Epub 2011 Jul 1. PMID: 21720923.

Moon SJ, Kwok SK, Park KS, Kim WU, Park SH, Kim HY. Simultaneous presentation of hemophagocytic syndrome and mesenteric vasculitis in a patient with systemic lupus erythematosus. Mod Rheumatol. 2011 Jun;21(3):330-3. doi: 10.1007/s10165-010-0401-8. Epub 2011 Jan 13. PMID: 21229376.

Carvalheiras G, Anjo D, Mendonça T, Vasconcelos C, Farinha F. Hemophagocytic syndrome as one of the main primary manifestations in acute systemic lupus erythematosus--case report and literature review. Lupus. 2010 May;19(6):756-61. doi: 10.1177/0961203309354906. Epub 2009 Dec 21. PMID: 20026522.

Takizawa Y, Maruyama J, Setoguchi K. Cyclosporin rapidly diminished a severe lupus-associated macrophage association syndrome refractory to pulsed methylprednisolone and intravenous immunoglobulin. Scand J Rheumatol. 2009 Nov-Dec;38(6):492-4. doi: 10.3109/03009740902971221. PMID: 19922027.

Horai Y, Miyamura T, Takahama S, Sonomoto K, Nakamura M, Ando H, Minami R, Yamamoto M, Suematsu E. Influenza virus B-associated hemophagocytic syndrome and recurrent pericarditis in a patient with systemic lupus erythematosus. Mod Rheumatol. 2010 Apr;20(2):178-82. doi: 10.1007/s10165-009-0241-6. Epub 2009 Nov 7. PMID: 19898920.

Taki H, Shinoda K, Hounoki H, Ogawa R, Hayashi R, Sugiyama E, Tobe K. Presenting manifestations of hemophagocytic syndrome in a male patient with systemic lupus erythematosus. Rheumatol Int. 2010 Jan;30(3):387-8. doi: 10.1007/s00296-009-0932-5. Epub 2009 Apr 19. PMID: 19381636.

Kampitak T. Fatal Kikuchi-Fujimoto disease associated with SLE and hemophagocytic syndrome: a case report. Clin Rheumatol. 2008 Aug;27(8):1073-5. doi: 10.1007/s10067-008-0902-4. Epub 2008 May 9. PMID: 18465190.

Takahashi N, Naniwa T, Banno S. Successful use of etanercept in the treatment of acute lupus hemophagocytic syndrome. Mod Rheumatol. 2008;18(1):72-5. doi: 10.1007/s10165-007-0006-z. Epub 2007 Dec 27. PMID: 18161002.

Sakai H, Otsubo S, Miura T, Iizuka H. Hemophagocytic syndrome presenting with a facial erythema in a patient with systemic lupus erythematosus. J Am Acad Dermatol. 2007 Nov;57(5 Suppl):S111-4. doi: 10.1016/j.jaad.2006.11.024. PMID: 17938019.

Kwon CM, Jung YW, Yun DY, Kim HD, Cho HS, Hong YH, Choi JH, Lee CK. A case of acute pericarditis with hemophagocytic syndrome, cytomegalovirus infection and systemic lupus erythematosus. Rheumatol Int. 2008 Jan;28(3):271-3. doi: 10.1007/s00296-007-0401-y. Epub 2007 Jul 12. PMID: 17624532.

Romanou V, Hatzinikolaou P, Mavragani KI, Meletis J, Vaiopoulos G. Lupus erythematosus complicated by hemophagocytic syndrome. J Clin Rheumatol. 2006 Dec;12(6):301-3. doi: 10.1097/01.rhu.0000249897.14441.c6. PMID: 17149063.

Hagiwara K, Sawanobori M, Nakagawa Y, Sato T, Akiyama O, Takemura T. Reactive hemophagocytic syndrome in a case of systemic lupus erythematosus that was diagnosed by detection of hemophagocytosing macrophages in peripheral blood smears. Mod Rheumatol. 2006;16(3):169-71. doi: 10.1007/s10165-006-0473-7. PMID: 16767556.

Qian J, Yang CD. Hemophagocytic syndrome as one of main manifestations in untreated systemic lupus erythematosus: two case reports and literature review. Clin Rheumatol. 2007 May;26(5):807-10. doi: 10.1007/s10067-006-0245-y. Epub 2006 Mar 31. PMID: 16575493.

Kawashiri S, Nakamura H, Kawakami A, Ida H, Izumi Y, Tamai M, Origuchi T, Moriuchi M, Moriuchi H, Eguchi K. Emergence of Epstein-Barr virus-associated haemophagocytic syndrome upon treatment of systemic lupus erythematosus. Lupus. 2006;15(1):51-3. doi: 10.1191/0961203306lu2247cr. PMID: 16482747.

Takahashi K, Kumakura S, Ishikura H, Murakawa Y, Yamauchi Y, Kobayashi S. Reactive hemophagocytosis in systemic lupus erythematosus. Intern Med. 1998 Jun;37(6):550-3. doi: 10.2169/internalmedicine.37.550. PMID: 9678692.

Plavsic A, Miskovic R, Jovanovic D, Karic U, Jovicic Z, Radovic S, Drazic A, Dasic A, Arandjelovic S. Life-Threatening Macrophage Activation Syndrome in Pregnancy: First Manifestation of SLE Induced by Parvovirus B19. Int J Mol Sci. 2025 Jun 4;26(11):5406. doi: 10.3390/ijms26115406. PMID: 40508215; PMCID: PMC12155505.

Lin N, Xu L, Dai Q. Variations in Macrophage Activation Syndrome-associated Cardiac Diseases: A Report on Two Cases. Iran J Immunol. 2023 Mar 14;20(1):135-143. doi: 10.22034/iji.2023.93355.2218. PMID: 36934324.

Guo R, Ye S, Gu L. Recurrent macrophage activation syndrome in patients with refractory systemic lupus erythematosus treated with emapalumab: a case report. Hong Kong Med J. 2025 Oct;31(5):387-390. doi: 10.12809/hkmj2512876. PMID: 41111353.

**Supplementary Table S9.** Results of the systematic literature case retrieval of reports describing previous cases of SLE-MAS.

| **Variable** | **N** | **Results** | **Variable** | **N** | **Results** |
| --- | --- | --- | --- | --- | --- |
| **Demographic variables** |  |  | **HScore** | 73 | 181 (137-222) |
| Female sex | 101 | 76 (75) | **MAS treatment** |  |  |
| Age at SLE onset | 94 | 27 (21-38) | Steroid | 102 | 101 (99) |
| Age at MAS | 101 | 30 (25-40) | Steroid pulses | 102 | 83 (81) |
| Death | 102 | 7 (6.9) | Cyclophosphamide | 102 | 16 (16) |
| Death due to MAS | 102 | 7 (6.9) | IVIG | 102 | 32 (31) |
| **MAS features** |  |  | CNI | 102 | 28 (27) |
| Fever | 102 | 100 (98) | Rituximab | 102 | 9 (8.8) |
| Hemorrhage | 102 | 12 (12) | PLEX | 102 | 9 (8.8) |
| CNS dysfunction | 102 | 23 (23) | Anakinra | 102 | 10 (9.8) |
| Hepatomegaly | 102 | 21 (21) | Etoposide | 102 | 7 (6.9) |
| Splenomegaly | 102 | 26 (25) | **SLE clinical features** |  |  |
| Lymphadenopathy | 102 | 39 (38) | Mucocutaneous SLE | 94 | 68 (72) |
| Hemophagocytosis | 99 | 75 (76) | Arthritis | 95 | 46 (48) |
| WBC | 94 | 2070 (1250-3200) | Nephritis | 95 | 24 (25) |
| Hb | 92 | 8.5 (7.25-10.5) | Pericarditis | 95 | 18 (19) |
| PLT | 96 | 80 (58-101.5) | Seizures | 96 | 2 (2.1) |
| AST | 77 | 163 (84-406) | Direct Coombs test | 36 | 21 (58) |
| ALT | 76 | 108.5 (58-216.5) | Leukopenia | 93 | 59 (63) |
| Creatinine | 39 | 1.11 (0.78-1.64) | Thrombocytopenia | 95 | 87 (92) |
| CRP | 60 | 38 (11-79) | Low C3 | 88 | 77 (88) |
| Fibrinogen | 54 | 1.9 (1.31-2.88) | Low C4 | 83 | 56 (67) |
| Ferritin | 99 | 4500 (1878-9560) | ANA | 98 | 98 (100) |
| Triglycerides | 73 | 300 (241-399) | anti-dsDNA | 97 | 74 (76) |
| C3 | 71 | 37 (26-64) | anti-RNP | 96 | 32 (33) |
| C4 | 64 | 7 (3-12) | anti-Sm | 96 | 39 (41) |
| Any infection | 102 | 28 (27) |  |  |  |

**Supplementary Figure S1**. Stability of LDA coefficients assessed by non-parametric bootstrap resampling (1000 resamples with replacement; linear discriminant analysis refitted on each resample). Bars represent the mean LD1 weight for each variable across bootstrap resamples, with error bars denoting the 95% bootstrap confidence interval; pink bars indicate variables favoring classification as SLE-MAS, blue bars indicate variables favoring classification as SD-MAS. Variables are ordered by mean LD1 weight.
